# An online evidence-based dictionary of common adverse events of antidepressants: a new tool to empower patients and clinicians in their shared decision-making process

**DOI:** 10.1186/s12888-024-05950-6

**Published:** 2024-07-25

**Authors:** James S. W. Hong, Edoardo G. Ostinelli, Roya Kamvar, Katharine A. Smith, Annabel E. L. Walsh, Thomas Kabir, Anneka Tomlinson, Andrea Cipriani

**Affiliations:** 1 Department of Psychiatry, University of Oxford, Oxford, UK; 2 Oxford Precision Psychiatry Lab, NIHR Oxford Health Biomedical Research Centre, Oxford, UK; 3 The McPin Foundation, London, UK; 4 Oxford Health NHS Foundation Trust, Warneford Hospital, Oxford, UK

**Keywords:** Adverse events, Harms, Antidepressants, Depression, Co-design, User-centred design, Lived experience, Personalised care, Digital psychiatry, Shared decision-making

## Abstract

**Background:**

Adverse events (AEs) are commonly reported in clinical studies using the Medical Dictionary for Regulatory Activities (MedDRA), an international standard for drug safety monitoring. However, the technical language of MedDRA makes it challenging for patients and clinicians to share understanding and therefore to make shared decisions about medical interventions. In this project, people with lived experience of depression and antidepressant treatment worked with clinicians and researchers to co-design an online dictionary of AEs associated with antidepressants, taking into account its ease of use and applicability to real-world settings.

**Methods:**

Through a pre-defined literature search, we identified MedDRA-coded AEs from randomised controlled trials of antidepressants used in the treatment of depression. In collaboration with the McPin Foundation, four co-design workshops with a lived experience advisory panel (LEAP) and one independent focus group (FG) were conducted to produce user-friendly translations of AE terms. Guiding principles for translation were co-designed with McPin/LEAP members and defined before the finalisation of Clinical Codes (CCs, or non-technical terms to represent specific AE concepts). FG results were thematically analysed using the Framework Method.

**Results:**

Starting from 522 trials identified by the search, 736 MedDRA-coded AE terms were translated into 187 CCs, which balanced key factors identified as important to the LEAP and FG (namely, breadth, specificity, generalisability, patient-understandability and acceptability). Work with the LEAP showed that a user-friendly language of AEs should aim to mitigate stigma, acknowledge the multiple levels of comprehension in ‘lay’ language and balance the need for semantic accuracy with user-friendliness. Guided by these principles, an online dictionary of AEs was co-designed and made freely available (https://thesymptomglossary.com). The digital tool was perceived by the LEAP and FG as a resource which could feasibly improve antidepressant treatment by facilitating the accurate, meaningful expression of preferences about potential harms through a shared decision-making process.

**Conclusions:**

This dictionary was developed in English around AEs from antidepressants in depression but it can be adapted to different languages and cultural contexts, and can also become a model for other interventions and disorders (i.e., antipsychotics in schizophrenia). Co-designed digital resources may improve the patient experience by helping to deliver personalised information on potential benefits and harms in an evidence-based, preference-sensitive way.

**Supplementary Information:**

The online version contains supplementary material available at 10.1186/s12888-024-05950-6.

## Background

Depression is a leading cause of mental illness, disability and reduced quality of life [1], with over seven million people in the UK alone being treated yearly with antidepressants [2]. On average, antidepressants reduce the severity of depressive symptoms [3], but adherence to these medications can be reduced by the occurrence of adverse events (AEs, defined as unwanted events or symptoms that may or may not be caused by the treatment) [4, 5]. Indeed, up to one-third of patients in randomised controlled trials (RCTs) discontinue their antidepressants due to AEs [3]. An improved understanding of potential medication-related harms and individual factors affecting the risk of harms could help to better tailor antidepressant usage during treatment whilst incorporating patient preferences [6]. However, for patients to express their preferences (e.g., about AEs they want to avoid) and for clinicians to collect and act on this information, both parties need to understand exactly what the distinct AEs refer to.

As mandated by regulatory agencies, AEs in RCTs are commonly reported in clinical studies using the Medical Dictionary for Regulatory Activities (MedDRA) [7], an international database for registering and classifying AEs used by industry and trialists. In MedDRA, each AE term generates a representative term, which sits within one or more hierarchies of terms incorporating disorder, organ and system categories [7]. This five-tiered hierarchy is arranged from the most specific (the Lowest Level Terms (LLTs), consisting of terms that correspond to the original reports) to the most broad, the System Organ Classes (SOCs), which consist of terms related to body systems or aetiologies [8]. MedDRA was developed by the International Council for Harmonisation of Technical Requirements for Pharmaceuticals for Human Use for drug safety monitoring and is widely used for AE data processing [9]. Despite this wide acceptance, this reporting system uses an industry-tailored language and makes it difficult for patients and clinicians to use this information for shared decision-making (SDM) in clinical practice.

Patient-centred care and research could help produce results that are more relevant to patients’ priorities for their healthcare. An example of this can be found in the field of oncology, where researchers and people with lived experience co-created the Patient-Reported Outcomes version of the Common Terminology Criteria for Adverse Events (PRO-CTCAE) to improve the validity, temporal precision, data completeness and meaningfulness of symptomatic AE reports [10–12]. The PRO-CTCAE was designed to complement the clinician-focused CT-CAE in generating patient-reported safety and tolerability data and was shown to be valid and reliable in assessing patient-reported symptomatic harms in a diverse group of patients in cancer trials [13]. A similar system in mental health trials could help to bridge the gap between research and practice by integrating patients’ experiences. For instance, the concept of life engagement, which includes well-being, quality of life and social involvement, may more meaningfully capture outcomes that matter to patients with mental health disorders [14], but implementation in clinical research and practice would require a concerted effort to co-design outcome measures with patients and evaluate their reliability and validity. Importantly, because of disorder-specific differences in symptomatic experiences, interventions and biological mechanisms [15, 16], a system that is tailored to the needs of mental health patients would require collaborative work with people with relevant lived experience.

Developing a common language of AEs, accessible to patients and clinicians, would be a critical first step in moving towards a patient-centred safety and tolerability system to address the unmet needs of clinical research. Such a system could improve transparency, communication and alignment of research output and goals between patients, clinicians and researchers. Although resources on the language and definitions of AEs of antidepressants exist (e.g., [17, 18]), there is little evidence of collaboration with people with lived experience or evaluation of their impact on SDM to maximise their usefulness, acceptability and accessibility. A freely accessible, patient-friendly dictionary of AEs of antidepressants could be an effective tool to access information about harms and make shared decisions in routine care.

Therefore, in this study we aimed to co-design an online dictionary of AEs of antidepressants with experts by experience, to enable patients and clinicians to have a shared understanding of AE terms and for patients to express meaningful preferences about specific AEs. Through a series of workshops and a focus group, we defined the AEs of antidepressants using data extracted from clinical trials of antidepressants and obtained insights into the ease of use, usefulness and acceptability of this new tool.

## Methods

### Clinical trials data, MedDRA and assignment of clinical codes

**Fig. 1:**
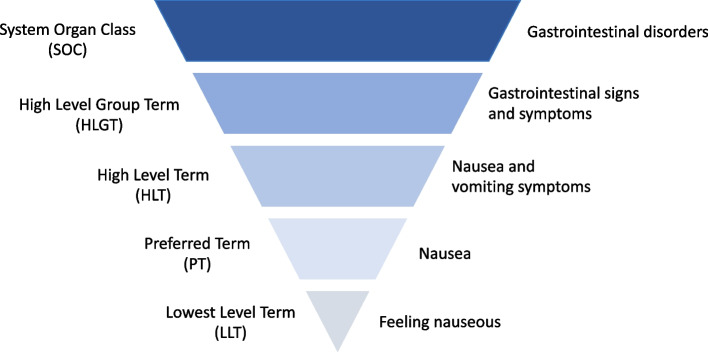
Example MedDRA hierarchy, from SOC (most general) to the LLT (most specific) adapted from [7]

AE terms were extracted from an existing pool of 522 RCTs, including published and unpublished reports [3]. The term for each AE was initially extracted as reported in the original paper (e.g., ‘abdominal pain upper’ or ‘stomach ache’ were extracted as different terms). We then manually searched for corresponding MedDRA terms which are arranged hierarchically (see Appendix, S1 for co-designed background information on MedDRA; see Fig. 1 for an example of the MedDRA hierarchy, adapted from the MedDRA website [7]).

In MedDRA, each of the lowest-level terms (LLTs: the most specific terms for AEs) involves at least one Preferred Term (PT), High Level Term (HLT), High Level Group Term (HLGT) and System Organ Class (SOC) combination. For this study, where more than one HLT was available, at least two clinically trained researchers (JSWH, EGO, AT, KAS, AC) discussed which HLT would be most clinically relevant to our field of interest (i.e., depression and antidepressants). Duplicate LLTs (e.g., ‘anaemia’ and ‘anemia’) and LLTs with the same PT, HLT, HLGT and SOC were merged. We then selected the MedDRA term that best represented a specific AE concept in terms of specificity and clinical relevance as a Clinical Code (CC) and listed the collected unified terms in the dictionary of AEs of antidepressants.

### Patient and public involvement, engagement and participation (PPIEP)

**Fig. 2:**
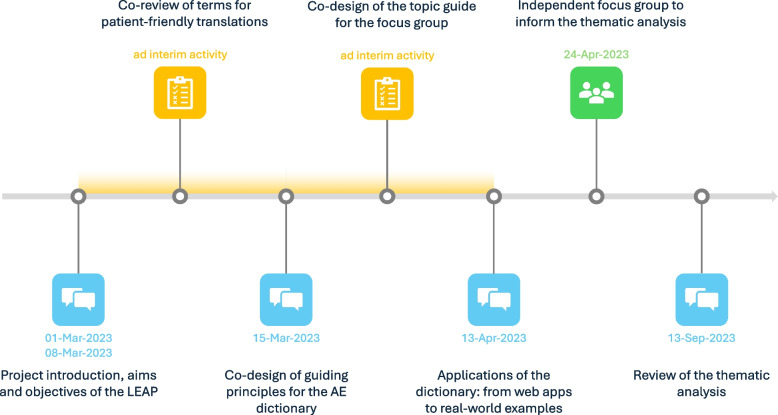
Timeline of co-design with lived experience experts. AE: adverse events, LEAP: Lived Experience Advisory Panel

Four 2-h workshops were conducted remotely with a Lived Experience Advisory Panel (LEAP), consisting of eight members of the public recruited nationally by the McPin Foundation (RK, AELW, TK). Inclusion criteria for the LEAP included: > 18 years of age, living in the UK, and past or current antidepressant usage for the treatment of depression, with capacity to consent to involvement. Each session was co-led by at least one clinical researcher (JSWH, KAS) and one person with lived experience from the McPin Foundation (RK, AELW). For the schedule of engagement with lived experience experts, see Fig. 2.

#### Co-designing a patient-friendly, online dictionary of adverse events

Based on iterative feedback from the LEAP and McPin team, CCs were translated into patient-friendly terms. The LEAP also developed guiding principles for creating patient-friendly translations. This was done by first inviting the LEAP to reflect on what was important to them in their individual translations of the CCs. This reflective process took place between workshops. In-session, they were then asked to elaborate on what were the key reasons for specific translations and factors that were important to them in approaching the translation task. Their thoughts were collated by a member of the McPin team (RK) and organised into three key guiding principles for the translation of terms. These principles, McPin/LEAP and clinician feedback, and feedback from an independent focus group (in domains including but not limited to content, format and accessibility) informed the development of the dictionary in a web-based form. Website development was done in collaboration with professional web developers.

#### Focus group on adverse events

Focus group participants (independent to the LEAP) were recruited by the McPin team to explore patients’ understanding of the meaning, importance and interpretation of AEs in their own lives. The eligibility criteria were the same as for the LEAP and recruitment was through purposive sampling (electronic advertisements were distributed through McPin’s network of people with lived experience). The purpose of the focus group was to draw upon the depth of the participants’ lived experience to inform our co-design of the dictionary. A participant information sheet, pre-session reading about the purpose of our research, aims of the focus group and relevant terms (AEs versus adverse reactions (ARs)), and topic guide (Appendix, S2-S4) were created in collaboration with the LEAP. The topic guide included questions on the experience of AEs when taking antidepressants, differentiating AEs and ARs (AEs are unwanted events/symptoms that may or may not be related to the treatment, whereas ARs are unwanted events/symptoms resulting from the treatment; see Appendix S3 for examples), impact on their lives, how they sought information about AEs/ARs and their experience of discussing AE/ARs with their clinicians. The two-hour session was conducted remotely and co-facilitated by members of the McPin Foundation (RK) and clinical team (KAS). Additional members were available for support of focus group participants and technological issues (AELW) or clinical queries (JSWH). Ahead of the meeting, written consent was obtained from all participants to record and store the video/audio output and demographic information for inclusion in publications in a secure cloud software to which only the research team had access. Consent was verbally re-confirmed by all participants who joined the online meeting. An anonymised transcript was produced for qualitative analysis using the Framework Method [19] conducted independently by at least two researchers (JSWH, KAS, EGO). Disagreements in coding and theme development during qualitative analysis were adjudicated internally.

The Research Governance, Ethics and Assurance Team at the University of Oxford deemed that ethical approval and University sponsorship were not required for this study.

## Findings

### MedDRA coding and clinical codes

A total of 736 AE terms were identified from 522 RCTs of antidepressants. After MedDRA coding and grouping into clinically relevant CCs, an initial list of 97 CCs was produced by the team. Through iterative work with the LEAP and focus group, we incorporated feedback suggesting a balance of breadth, specificity, generalisability, and patient-understandability and acceptability. Examples of the extracted AE terms, CCs and the rationale for co-translation with lived experience experts are available in Table 1. Additional definitions were created for each CC for patients to refer to, including links to existing online resources (e.g., NHS and government websites). Some CCs in the initial list were expanded based on PPIEP/clinician feedback suggesting that they contain more than one subjectively or clinically important AE concept (see Table 1). This resulted in a final list of 187 patient-friendly CCs comprising the dictionary of AEs (Appendix, S5) for the development of a website to host the online version of the dictionary, accessible to clinicians and patients (https://thesymptomglossary.com/).

**Table 1:** Examples of AE terms, CCs, and rationales for translation

Example number	Extracted AE terms	Clinical Code	Rationale for translation
1	‘Asthenia’ ‘Asthenia/fatigue’ ‘Daytime fatigue’ ‘Lassitude’ ‘Lethargy’ ‘Tiredness’…	‘Tiredness/weakness’	Combining related terms into an umbrella term that is more understandable
2	‘Gait disturbance’	‘Disturbance in walking/gait’	Making a term more lay friendly through rearrangement
3	‘Dyskinesia’	‘Involuntary writhing movements’	Making a term more lay friendly through descriptive clarification
4	‘Ejaculation disorder’ ‘Male orgasmic disorder’	‘Ejaculation problems’	Combining related terms as well as choosing an alternative word for ‘disorder’ to reduce stigma and enhance acceptability
5	‘Bradycardia’	‘Heart rate decreased (lower than the normal range)’, accompanied by an additional statement clarifying the medically accepted minimum (60 beats per minute)	Making a term more lay friendly and clarifying the medically defined threshold for a slow heart rate
6	‘Anxiety’ ‘Nervousness’ ‘Feeling of panic’ ‘Tension’…	Initially merged under ‘Anxiety’, but after later feedback, the final CCs included both ‘Anxiety’ and ‘Feeling of panic’	Feedback suggesting an important subjective and clinical difference between (general) ‘anxiety’ and ‘feeling of panic’ warranted the inclusion of separate CCs in the dictionary

*AE Adverse event, CC Clinical code*

### Guiding principles for translating adverse event terms

During the process of patient-friendly translation of terminology, the lived experience group members identified three guiding principles:

1. Mitigating stigma: specifying the context and explanation
  LEAP members highlighted the importance of avoiding stigmatising language in the description of AEs, whilst acknowledging that there may be a valid reason or meaning in such terms within a medical context. For example, ‘disorder’ or ‘abnormal’ could be considered stigmatising but are sometimes required for specific reasons (for example an ‘abnormal blood test’ for an individual implies a quantitative result that is outside a biomedically accepted range of normal values). The LEAP highlighted how a patient-friendly dictionary could provide alternative terms and/or further details on their medical context to lower the chances of stigmatisation.
2. Levels of comprehension
  LEAP members suggested that there is no universal definition of what ‘lay’ means, highlighting that there are multiple levels of comprehension in the general population. It was suggested that this should be reflected in the dictionary of AEs, thus having multiple levels of lay language and accessibility. For instance, this could be done by considering the personal, educational and professional backgrounds of the audience, those who speak English as a second language, their general comprehension of the language and providing explanations at different levels to accommodate for this range.
3. Relevance for different users: clarity and accuracy of meaning
  LEAP members suggested that there is no universal definition of what ‘lay’ means, highlighting that there are multiple levels of comprehension in the general population. It was suggested that this should be reflected in the dictionary of AEs, thus having multiple levels of lay language and accessibility. For instance, this could be done by considering the personal, educational and professional backgrounds of the audience, those who speak English as a second language, their general comprehension of the language and providing explanations at different levels to accommodate for this range.

### Thematic analysis: insights into the adverse experience from a focus group

The demographics of the participants of the focus group are available in Appendix, S6. A summary of thematic results is below, with selected quotes in Table 2 (see Appendix, S7 for more details).

**Table 2:** Quotes relating to themes from the focus group

Theme	Quotes^a^
**Theme 1** **The Concept of Adverse Events (AEs)/Adverse Reactions (ARs) and Information about AEs of Antidepressants**	**Language of the concept of adverse events and reactions**
	Confusion regarding meaning: “I was also finding that through this whole conversation that I’ve actually been a bit confused about whether we were talking about side effects or events…” (Participant 8)
	Real-world relevance is important: “I supposed the word adverse events, I don’t know if that’s going to mean a lot of things to quite a lot of people…so most people would talk about side effects…” (Participant 5)
	**Details about adverse events (AEs)**^b^
	General need for more information: “I think the more information about those real details about side effects, for different people with different lives and needs, you would be able to compare and contrast much more.” (Participant 4)
	Breadth and specificity of terms used to describe AEs: “You can go from feeling nervous to having feelings of panic. I think they’re very different feelings. I think it’s actually really useful to have quite a few different words to describe what that might feel like.” (Participant 8)
	Symptom-based versus non-symptom-based (emotional and functional impact of AEs): “it feels like what people are describing are quite matter of fact” (Participant 8), referring to description/characterisation of specific symptoms; “So most people would talk about side effects and also for me, it would be more like life change as well which could include a life event or a change in circumstances rather than maybe adverse events.” (Participant 5)
	Likelihood: “if you understand the likelihood or the seriousness of it, it would also be one more thing you can bring to the GP so that you can be more of an equal in that shared decision making about your own health.” (Participant 12); frequency of specific AEs: “I’ve also had really vivid dreams every night since I’ve been on Sertraline” (Participant 11); prevalence or ‘commonness’: “side effects which aren’t very common to experience” (Participant 8)
	Dose-dependence: “Whether there’s anything around that as well, about dosage, I don’t know whether any of that will be included because that can definitely make a change. So long as you’re okay on the medication but once you start to up the dosage, the side effects could be a lot more.” (Participant 5)
	Duration and Severity: “I think it’s really helpful to know those things, especially when you’re starting the medication. I think it really helped me to know the intensity and how long it would last for so that I would stick with the medication. If I hadn’t known, I don’t know if I would have wanted to continue with that. So yes, it was really beneficial for me.” (Participant 11)
	Overall impact of AEs: “Is Citalopram the entry level antidepressant that has the fewest side effects and they start you off on that? Who knows.” (Participant 8)
	Expectedness versus unexpectedness: “…to understand what might be considered a normal symptom” (Participant 12); “That’s what just feels so weird about all of this, is that some of this stuff feels so random and you wouldn’t anticipate it to be impacted but it is.” (Participant 8)
	**Adverse events versus adverse reactions**
	Rationalising one’s experience of AE vs AR: “if I know why something is happening then I can maybe rationalise it and think, “I can’t hear things properly or everything because this is what is happening to my brain. Is that rational or is that just this weird, kooky side effect?” (Participant 8)
	Difficulty in making AE-AR interpretation:
	• *Irregular symptoms*: “[Migraines] could come every couple of months. So it’s thinking, “Is it happening now because I’ve always had it or is it happening now because of the medication?” (Participant 12)
	• *Overlap with depressive symptom*: “[disturbed sleep was a] symptom of my illness when I wasn’t medicated. So again, sometimes it could be a sign that my illness is bad at that time but also I think the medication is having an effect.” (Participant 4)
	• *AE or therapeutic response?:* “I wasn’t feeling low but also I wasn’t feeling my bubbly self. There was just numbness which at the time I was like, “This feels good,” but actually it wasn’t real life.” (Participant 6)
**Theme 2** **Shared decision-making (SDM) (between healthcare professionals and patients in antidepressant treatment of depression)**	**Clarifying the personal therapeutic goal for SDM**
	Establishing the individual’s therapeutic goal: “I’m much more willing to accept this symptom or the medication not working in this way as long as this part is resolved,” because there’s a lot of things you’re hoping to achieve from these medications and not just one thing.” (Participant 12); “…it’s really important to listen to each person…” (Participant 12)
	**Interactions with the healthcare professional (HCP) and SDM**^c^
	Teamwork (MDT) and organising multiple information sources “I’m lucky that my GP is specifically interested in overprescribing and withdrawal.” (Participant 8); “…really important role for pharmacists attached to GP surgeries – I am lucky that my GP surgery have 2 full time pharmacists…they do all the reviews and supported me with increases and decreases in medication doses” (Zoom chat)
	Accuracy of the Content: “It was amazing because it was exactly how [the GP] said it would work. So yes, I had a very good experience in that sense.” (Participant 6)
	Quality of communication: “[the clinician] had sold it in a really blasé way, ‘You might feel a little bit wobbly. You might feel a bit thirsty,’ it was totally played down.” (Participant 8)
	Quality of the relationship: “…whether you’ve got a longer term relationship with a GP…I, for the first time in my life, have got one named GP that I see all the time and that’s really helped.” (Participant 8)
	^c^*See at the bottom of this Table for the variety of emotions experienced, during interactions in antidepressant treatment and AEs*
	**Perceived barriers or challenges to providing care based on SDM**
	Stigma: “I’d be worried about people feeling they have to be ‘bad enough’ and that feeds a feeling of stigma” (Zoom chat)
	‘Forced’ to adhere: “In my case, I found it so hard to withdraw that I’m just on a medication just because it’s easier to stay on it than to come off. That’s not the right place to be in…” (Participant 8)
	Time: “…being able to secure 20–25 min long appointments. That was really when we discussed the medication…I had to pay for [the private consultation]…” (Participant 9); “I’ve had a slightly different experience than [Name]. I’ve managed to find a GP that does have experience and interest and does give me time. But my last wait for my GP was over an hour so he runs behind. That’s how I get time with him, is because he is always running…” (Participant 8)
	Shift in healthcare system: “it’s so commonly prescribed there [in the USA] for the things that I was experiencing, that it seemed like a natural fit.” (Participant 9), but after moving to the UK, “during the pregnancy, it was really scrutinised that I was on this medication so there was constant advocating for myself to say that this was right, it is normal in the States…” (Participant 9)
	Dis/continuity of care: “…great when get the same GP and understands” (Zoom chat); “…if you were lucky enough to have [a discussion] with a doctor about which medication you might prefer.” (Participant 4)
	**SDM and enhancing patient autonomy**
	Informed choice: “if you, as the patient, one as the patient, is aware of the full impact of taking that medication, then you can make an informed decision about whether to take it.” (Participant 3)
	Self-learning and discovery: “…in the end, you have to learn for yourself. You learn, by stepping up through medication, often how sensitive you are or not to the effects.” (Participant 4)
**Theme 3** **Personalised care and facilitators, barriers and challenges to its delivery (Personalised care of people with depression; and facilitators (things that help), barriers (things that hinder) and challenges to delivering this care)**	**Understanding the patient as an individual**
	Being seen and understood as an individual: a clinician “might not know your situation or they might [not] know the medication or even what you’re dealing with very well” (Participant 12)
	Person-specific experience/tolerability: “I’ve not met another person whose had noise sensitivity or even some of the side effects I’ve described.” (Participant 8); “Some people can just stop taking the drugs and they’re fine. I’m doing it over six months because I’m so sensitive to it.” (Participant 8); “…the severity, again, it’s really personal, isn’t it…” (Participant 4)
	**Considering contextual factors around the AE experience to personalise care** a priori acceptability of specific AEs: being a “newlywed at that point so it was really important to have libido there” (Participant 9)
	Perception of a rational/acceptable benefit-risk ratio: “…there have been only two trials and they’ve been so small that there hasn’t been any conclusive evidence that it does harm the baby by any means…I know that I would be a lot worse off if I hadn’t taken it during the pregnancy.” (Participant 9)
	Optimal timing of starting antidepressant: “if you’ve got an interview next week, you might want to think about starting them the week after…even if you’re just prompted to consider when you might start taking them would be helpful.” (Participant 8); “So for example, if you’ve got a weekend at home, you could perhaps start the new [antidepressant]… I think it can really vary whether you get that advice or not, whether you can be in a safe, comforting space to try them.” (Participant 8)
	Considering the anticipated time course of clinical effects, the GP “signed me off from work. She said, ‘Because of that, I’m going to sign you off for a month.’” (Participant 6)
	**Considering the impact of depression on the person’s life and functional capacity to personalise care**
	Referring to the need for personalised support during treatment, “certain drugs might impact different ways of thinking” (Participant 8); “the knock-on impact that [adverse events have] on your day, if you’re also having to manage these things alongside daily life” (Participant 8)
	**Antidepressant history (history-specific memories) and personalising care by engaging with the patient’s identity**
	Centrality of ‘my’ or ‘their’ antidepressant and emotional exhaustion “I’ve been on sertraline and escitalopram, those are the ones I have experience with.” (Participant 12); “Actually many years ago I took sertraline for my nausea with it…” (Participant 5); “…had other things like amitriptyline which was pretty horrendous, blurred vision, metallic taste in your mouth. That certainly was a pretty horrendous side effects there…” (Participant 5)
	“…and so my history with taking the medication that I’m on currently is Wellbutrin in the States, also known as bupropion here…It’s been better than anything else I’ve tried but here in the UK, it’s often only prescribed for smoking cessation…I’ve experienced different things while taking it, however I would say the pros outweigh the cons.” (Participant 9)
	**Approaches to management, personalised versus non-personalised**
	Trial-and-error: “I mean I know lots of people’s experience is you kind of just get given something and you try it and if you can tolerate it then you probably just stay on it.” (Participant 4); “I’ve tried quite a few different antidepressants. The one I’m [on] now is one that seems to work quite well for me…” (Participant 5)
	Paternalistic care: The participant was told that she ‘[has] to take sertraline’ and ‘wasn’t given a choice even though [she] didn’t find it effective’ (Participant 12)
	Hope for a personalised approach: “I also discussed it with my therapist at the time to try and find different medications that might be a good fit but she wasn’t a psychiatrist so she couldn’t prescribe anything but we just discussed the different options out there.” (Participant 9)
**Theme 4** **Usefulness and acceptability of information in an online resource**	**Limitations of currently available resources on antidepressants and AEs**
	Partial availability of relevant information: “information [about duration and severity of AEs] is kind of there to a point but not really” (Participant 4)
	Need for support in translating information to communication/expression: “I can read and write, I can research things in theory, I have the tools at my disposal to make an informed choice but I didn’t feel like I could ask for something different” (Participant 8)
	**Important qualities of an online resource**
	Breadth: “I think there’s a lot of benefit to show the breadth of how that issue might present in an individual.” (Participant 8)
	Common language: “it’s very helpful to have language that Joe public would use. When I go and see my GP, I don’t talk about having gastroenter-, whatever it was, I can’t even say it let alone mention it to my doctor. I would probably go in and say I have constipation. A common language that most people use is obviously helpful when you’re talking about side effects or adverse events.” (Participant 3)
	Credibility: “that a GP might maybe respect you a bit more if you’re actually pointing to a resource and saying I’m experiencing tick, tick, tick, this, this, this for example.” (Participant 8)
	Ease of use: “me, I find that quite easy to go to the news and I quite like that visual thing. I think to be able to check something out about it and then click on other links but that’s just a personal thing, that would work quite well for me.” (Participant 5); “I think the less work people have to do to feel like what they’re experiencing is valid or real, and there’s a label that they can use or point to is better than them putting it on to the person that’s having those negative feelings, have to go and research or click on extra links and expand it all out.” (Participant 8)
	Balance of features: “unpack the pros and cons of having those extra words there.” (Participant 8)
	Visual format/presentation: “keep some idea of the severity of either, perhaps via the little icon or smiley or something, would be helpful.” (Participant 3); “I think it would be really nice to have that visual, almost like a grid of different medications, which ones have a likelihood of having certain effects to it.” (Participant 12)
	**Uses for an online resource**
	To engage in SDM: e.g., guided discussion: “…it is categories in a way that says this is what’s serious, this is what you need to talk to your GP…” (Participant 12)
	To seek information: “Something that’s a real slow burn might be better for some people but other people might…have the ability to take a big hit on lots of intensity but over a shorter period…I think that comparison around the length of time you’ll put up with these varying things would really help you…” (Participant 4)
	To validate experience: “But I still think a gauge, to know that for lots of people it’s this rough amount of time, at least you can keep the faith if you feel rubbish but you can see that for a lot of people it’s a long effect. I like it.” (Participant 4)
	**Information on pharmacology**
	“…a brief description within the dictionary, within the medication itself on how the body uses it, how it functions within the body might be useful to anticipate symptoms even if they’re not listed.” (Participant 12)

^a^Note that the participants are numbered up to 12, but the total number of participants in the focus group is 8 (4 out of the 12 were facilitators of the focus group)

^b^Refer to the full thematic analysis (Appendix) for details of adverse events not included here

^c^Feeling of having been heard: “[The clinician] was really good … She was very caring.” (Participant 6); Self-doubt: “I thought was it [i.e., starting to feel suicidal] the nature of the medication perhaps not working so well with me.” (Participant 5); Denial: “I was actually told that the things I was experiencing wasn’t related [to the antidepressant] but I knew it was.” (Participant 8); Feeling judged: “…it was just a clear cut, ‘No we cannot prescribe that for you because we don’t do that here. You’re not a smoker so why do you need this?” (Participant 9); Feeling overwhelmed: “…I was on the absolute cusp of coping emotionally each day. So then to also have all of these other physical things to manage was nearly impossible.” (Participant 8); Feeling prepared: “I think I was prepared for that because I had a long session about that [the side effects].” (Participant 11); Feeling unprepared: “I think I was totally naïve into the process of what starting this medication would be for me.” (Participant 8); Feeling worried: “My GP was mentioning the suicidal thoughts that might be occurring. So I was really worried about.” (Participant 11); Feeling uninformed: “I just feel like would I eat something that I didn’t know what was in it or didn’t know what it was going to do to me.” (Participant 8); Feeling forced into a choice: “when I first started antidepressants, I was breastfeeding and I wasn’t given a choice. They said, ‘You have to take sertraline,’ that was it. I wasn’t given a choice even though I didn’t find it effective.” (Participant 12)

### Clarifying the concept of AEs and their relevance to SDM

Participants acknowledged the importance of distinguishing between the concepts of AE and AR, but they expressed confusion about their meaning. For instance, participants often referred to ‘side effects’ (a term which some felt was more relevant than ‘adverse events’ or ‘adverse reactions’). In other cases, whilst acknowledging the importance of the conceptual distinction between AE and AR, they questioned the practical utility of these terms in clinical practice. Building on this, the participants emphasised the importance of the real-world relevance of terms that distinguish AEs versus ARs, as well as individual AEs (e.g., specific symptoms) when discussing within everyday clinical practice so that they can accurately and meaningfully express what matters to them.

Participants suggested that more detail such as symptom severity and duration of AEs (Table 2) are needed in SDM around antidepressants to help predict the impact on their function and quality-of-life. For instance, maintaining concentrating ability may be more of a priority for one participant and avoiding tremor for another, depending on occupation, lifestyle and individual preference. Furthermore, an individual may choose a short period of intense symptoms during medication dose changes, whereas another may have a lower threshold for tolerating symptom severity. These dimensions were important in (i) deciding to start, continue, switch or stop an antidepressant, (ii) rationalising and validating experience of an effect, (iii) making a judgement about causal attribution of a symptom and (iv) managing their expectations and behaviour during antidepressant treatment (influencing tolerability and adherence) (Table 2).

### From SDM, individual goals and preferences about AEs to personalised treatment

Many participants suggested that a primary reason for improving the clarity of concepts and details of AEs is to facilitate SDM in routine care. They highlighted the importance of the individual’s therapeutic goal which should incorporate preferences on AEs, and that these preferences vary between individuals in how they are ‘ranked’ in order of importance.

Participants discussed how these goals and preferences could be best elicited within SDM with their clinicians, and that the perceived overall quality of the healthcare interaction would depend on the accuracy of information, quality of communication and quality of the relationship (Table 2). Ultimately, efforts to improve SDM were perceived as efforts to promote individual autonomy in various forms (e.g., education, empowerment, advocacy). SDM was believed to be essential to personalised care because it provides a robust framework and process in which to collect, exchange and act on information, including preferences, to formulate an individualised decision about treatment.

### Personalised treatment incorporates multiple levels of data that are context-specific

Participants discussed how a key value of a longer-term and collaborative doctor-patient relationship was based on better personalisation. Personalisation requires that the appropriate dimensions of AEs are considered and meaningfully expressed (i.e., communicating preferences). Participants suggested that how these dimensions and preferences are considered may depend on the decision-making scenario (Table 2, e.g., starting, continuing, switching or stopping an antidepressant). They discussed how preferences may reflect several factors related to the acceptability of specific AEs, timing of decisions, the time course of effects and individual life contexts (Table 2). Personalisation was also conceived to involve helping make sense of and validate their experience.

### Available resources on the harms of antidepressants and suggestions for the new online dictionary resource

Participants felt that currently available sources of information were often incomplete and only partially helpful in SDM. They highlighted the need for more support in translating the content of information to better ways of communicating and discussing their goals and needs with their clinician.

Thoughts about desirable qualities of an online resource included: (i) breadth and specificity of AEs in an online resource, (ii) simple, accessible and inclusive language, (iii) credibility (reliability and robustness) of underpinning research, (iv) ease of use, (v) balance of features, and (vi) visual format of communication of benefit and harm information. Additionally, some participants felt that pharmacological information (such as the mechanisms of action of antidepressants or the way they are absorbed and metabolised by the body) would be useful for them to think through their own experience and try to rationalise the expected- versus unexpectedness of their AE/ARs. These qualities were not exhaustive but served as a starting point for the co-design of a user-friendly resource. An online resource with such qualities was envisioned to be useful for engaging in SDM with the clinician; seeking relevant, trustworthy, and easy-to-digest information; and validating the subjective reality of their unique experience of depression and antidepressant treatment. An option to submit feedback in real-time was also advised for inclusion in the website, as well as customisable templates to empower patients to communicate their AEs to clinicians.

### A prototype of an online dictionary of AEs of antidepressants

**Fig. 3:**
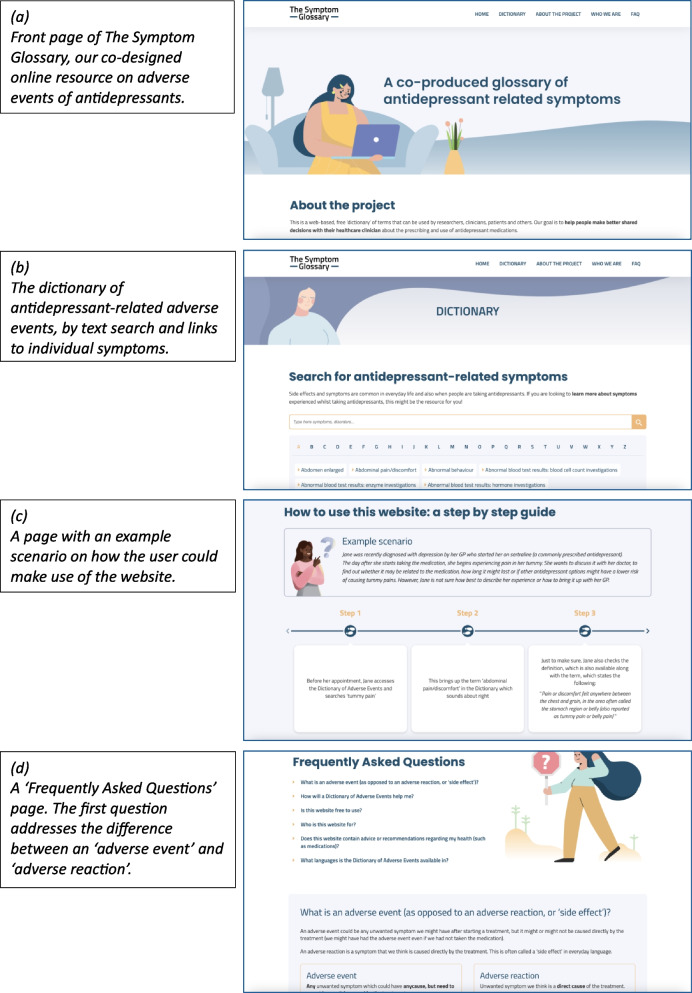
**a**-**d** The Symptom Glossary website (https://thesymptomglossary.com)

A pilot webpage hosting the online dictionary of AEs was created (Fig. 3). Incorporating PPIEP feedback, we aimed to make the AE search process more flexible by including both an open search field, similar to commonly used online search engines, as well as a list of terms (CCs) derived from the translation (examples in Table 1). Reflecting both sides of the SDM process, we included a dictionary from both patient and clinician perspectives. Furthermore, we included definitions/descriptions of the CCs and links to other freely available (e.g., NHS) resources; a Frequently Asked Questions section including relevant definitions on broader concepts (AE versus AR); and a step-by-step guide on how one might use the website.

## Discussion

In this project we co-designed an online evidence-based dictionary of antidepressant-related AEs and identified factors important to the preliminary feasibility, usefulness and acceptability of the tool. Incorporating these factors may have the potential to enhance communication of expectations and previous experiences of AEs associated with antidepressants during SDM.

Our work on translating 736 AE terms to 187 CCs incorporated feedback on the important balance of breadth, specificity, generalisability, patient-understandability and acceptability. Efforts involved capturing a comprehensive list of AE terms arising from unpublished and published data from RCTs [3], and coding decisions were made by the clinical/research and PPIEP members together. We were guided by co-designed principles for translation. The terms aimed to be accurate and understandable, but also nuanced such as to strengthen the therapeutic relationship rather than alienating or stigmatising individuals. We used multiple co-design approaches, including a series of workshops and a separate focus group as we progressed from coding using MedDRA [7] and the translation of AE terms to the development of the web-based dictionary.

Our work has some limitations. It was not designed to mitigate pre-existing limitations in antidepressant trial protocols which vary in their specificity in collecting/processing harms data [20–22] and historically have not included lived experience experts in their design [23]. The work cannot be considered an exhaustive list of all the various AEs that patients consider important in the real world (e.g., ‘brain zaps’, ‘zombie’), because the extracted AE terms were limited to those reported in RCTs. Furthermore, our dictionary gives no information about the specific AEs that are associated with specific antidepressants. Some areas of importance to the LEAP/focus group could not be explored in depth, such as harms-versus-benefit information on antidepressant usage in pregnancy/breastfeeding and specific symptom domains such as executive or cognitive dysfunction. Research into the impact of antidepressants during pregnancy is an under-researched area, despite studies showing an association of antidepressant usage during pregnancy with harms [24]. The consideration of harms and benefits of antidepressants in pregnancy requires a patient-centred approach to decision-making [25], which needs further research. Future work could explore the language of AEs, SDM and challenges unique to these populations, cultural adaptations of the tool [26], gather data on the real-world experience of patients, and aim to expand the diversity of the lived experience panel.

Our work revealed specific dimensions of AEs that matter to patients. These are important because empirical accuracy and precision in describing an experience are an important basis on which to form or express preferences essential to SDM and personalisation of treatment. Some dimensions, such as frequency and intensity, are widely used in clinical research [27]. Others, such as quality of life, treatment satisfaction, or functional improvement, are increasingly recognised for their importance in mental health research despite their variable adoption [28]. One reason for the lag in adoption of such measures may be the historically low engagement with people with lived experience [29], whose involvement is critical to developing reliable and valid patient-reported outcome measures (PROMs) that capture these outcomes. Other reasons include concerns about psychometric properties, costs, administrative/logistic challenges and interpreting PROMs [30, 31].

Concerning the definition of each distinct AE, it will be important to research and implement outcome measures that matter to patients. This is because individual preferences and values are impacted not only by the type of experience (represented here by CCs) but also by quantitative or qualitative aspects (dimensions) of each experience, and preferences may change depending on the individuals’ threshold of tolerability in relation to these outcome measures [32]. For instance, preference about ‘nausea’ or ‘headache’ may change depending on one or more dimensions, such as (anticipated or experienced) severity, duration, frequency and functional impact. To some individuals, some AEs may be unacceptable independently of what dimensions are being considered – they may prefer that the experience does not occur at all. A shared understanding of these dimensions, their communication within a collaborative process, and meaningful outcome measures will be critical in personalising treatment [6].

Our results show that patient preferences reflect the importance of various, often conflicting, goals and acceptable means by which to achieve or avoid certain effects during antidepressant therapy. The transfer of information requires expertise, quality of communication and a good clinician-patient relationship [33]. A tool to improve SDM could target one or more of these aspects. SDM is relevant to personalised care because it provides the optimal ground for parties to gather and act on relevant information to formulate a person-specific decision [34]. A tool to help with personalisation should help to make context-specific decisions, apply the best available evidence, and incorporate preferences [35].

Given the above, we created an online, evidence-based dictionary of AEs that addresses a key aspect of SDM, that is, to facilitate clarity and precision of what is being asked for from patients (in terms of AEs), what the patients refer to in describing adverse experiences, the acceptability of the language, and the meaningful expression of preferences. However, there are other aspects of SDM and personalised treatment to improve, which could be done at scale through digital tools [36]. An ongoing challenge in co-designing these tools will be to recognise the diversity of lived experience whilst maintaining focus on the relevant clinical population/phenotype, question and intended usage [37]. Additionally, research into harms will require greater rigour and transparency in evidence synthesis and communication [20–22]. This is important so that information can be understood by multiple stakeholders [38]. It will also be necessary to update our website so that it is informed by the latest research and keeps pace with advances in technology. Whilst a discussion of seriousness was not within the scope of our work which focused on common AEs [6], it may be important to update the website with uncommon or rare and/or serious AEs as well. Crucially, we aim to disseminate the website using multiple strategies incorporating social media, word-of-mouth, and patient-friendly newsletters, facilitated by lived experience groups internal to our NHS Trust as well as the existing network supported by the McPin Foundation.

Finally, our work highlights that the use of a SDM approach may improve the therapeutic alliance [39], and a strong therapeutic alliance may enhance the clinicians’ and patients’ ability to engage in SDM [40]. Because multiple trade-offs of various harms and benefits are required in antidepressant treatment, the effective communication of harms and benefits tailored to the individual is key to strengthening this alliance [41, 42]. However, it remains challenging to deliver patient-centred care that incorporates individual- and context-specific information. Participants highlighted tensions that exist between patients and clinicians in the language, interpretation of harms-versus-benefit trade-off, individual acceptability and therapeutic priorities. Patients want to navigate these tensions whilst remaining engaged as autonomous decision-makers in collaboration with their clinicians [43]. Rigorously developed tools, such as patient decision aids (PDAs), could enhance patient autonomy by increasing their ownership of and engagement in their care [44], whilst supporting clinicians to deliver personalised care [45]. PDAs have been associated with improved decision-making processes and decision quality, in general [46] and specific clinical populations (e.g., depression [47] and post-traumatic stress disorder [48]). The implementation of digital support tools could improve adherence [49] and, with appropriate training of clinicians and patients, the quality of services [50, 51]. Such tools should be co-designed with the people whose experiences are the subject of exploration and target of treatment to maximise their impact [52]. Recent innovations in digital psychiatry hold tremendous potential in mental health [53].

## Conclusions

Our study showed that multiple factors, including breadth, specificity, generalisability, understandability and acceptability of language, are important to patients in defining and expressing preferences about AEs in antidepressant treatment. AEs should also be characterised with respect to empirical dimensions that are meaningful to patients, which may require the research and development of novel PROMs. Finally, the effective consideration of antidepressant options in SDM may be facilitated by patient-friendly, accessible digital tools. Our free online dictionary may help empower patients to communicate their experience of AEs more accurately during their antidepressant treatment journeys. With a shared language, patients may more confidently engage in SDM with their clinicians. For clinicians, the dictionary may help them to enhance the patient experience by helping to deliver personalised harms-benefit information in an evidence-based and preference-sensitive way. Future iterations of the work should incorporate patient and clinician feedback on the online tool to improve its user-friendliness. The tool may also become a model for other interventions and disorders, such as antipsychotics in schizophrenia.

### Supplementary Information

Supplementary Material 1.

Supplementary Material 2.

Supplementary Material 3.

Supplementary Material 4.

Supplementary Material 5.

Supplementary Material 6.

Supplementary Material 7.

## Data Availability

The datasets used and/or analysed during the current study are available from the corresponding author on request.

## Abbreviations

AE: Adverse event
AR: Adverse reaction
CC: Clinical Code
CTCAE: Common Terminology Criteria for Adverse Events
HLGT: High-Level Group Term
HLT: High-Level Term
LEAP: Lived Experience Advisory Panel
LLT: Lowest-Level Term
MedDRA: Medical Dictionary for Regulatory Activities
PDA: Patient decision aid
PPIEP: Patient and public involvement, engagement and participation
PRO-CTCAE: Patient-Reported Outcomes version of the Common Terminology Criteria for Adverse Events
PROM: Patient-reported outcome measure
PT: Preferred Term
RCT: Randomised controlled trial
SDM: Shared decision-making
SOC: System Organ Class
