# Supplementary material for: An online evidence-based dictionary of common adverse events of antidepressants: a new tool to empower patients and clinicians in their shared decision-making process
Source: BMC Psychiatry. 2024 Jul 25;24:532. doi: 10.1186/s12888-024-05950-6 (PMC11270875; doi:10.1186/s12888-024-05950-6)
Supplement: Supplementary file 1 — Supplementary Material 1. [file 12888_2024_5950_MOESM1_ESM.docx]

**Creating a free online dictionary to explain and present the unwanted effects of antidepressant medications in a clear and accessible way**

**(Plain English version)**

All medications have unwanted effects. For example, a dry mouth. Some effects are more serious than others. Within a research study these are known as ‘adverse events’. These adverse events may, or may not be due to the medication being studied. But it is important to record everything in case it is due to the medication.

Firstly, we conducted a search of the published research of antidepressants for depression where adverse event information had been reported. We then made a list of all the adverse events, in the exact form that they were reported. For example, “abdominal pain upper” and “abdominal pain” would be extracted as two different terms, and two different adverse events. This is because two similar, but different wordings were used.

Next, we used an existing way of classifying adverse events, called the Medical Dictionary for Regulatory Agencies (MedDRA) – [www.meddra.org](http://www.meddra.org). MedDRA is a database developed to provide a *single* ‘standard’ wording for each adverse event that can be used internationally. This is very important because as we have seen, there can be different ways of referring to the same adverse event.

In MedDRA everything is organised into five levels arranged from very specific to very general. Figure 1 gives an example, for the term ‘feeling queasy’. When you put in the term “feeling queasy” into the MedDRA database, the system generates a new term at each of these five different levels.


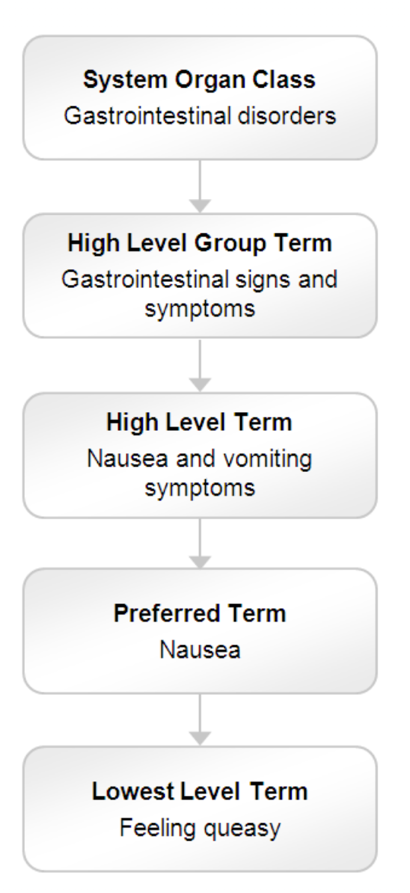


*Figure 1. Example of MedDRA hierarchy, for the lowest level term ‘feeling queasy’ (Source:* [*https://www.meddra.org/how-to-use/basics/hierarchy*](https://www.meddra.org/how-to-use/basics/hierarchy)*)*

Whilst MedDRA is very useful for the pharmaceutical, research community, and the NHS it is not always meaningful for people who take antidepressants, with terms that are very medical and not often relevant to people’s everyday experiences. Additionally, we found that there were hundreds of different ways in which adverse events were reported.

To make sense of the bewildering variety of terms we need a good way of

- Explaining adverse events in as clear a way as possible so everyone understands what they mean
- Organising similar adverse events into ‘groups’ – which we call ‘clinical codes’

We have tried to make sure that the groups or ‘clinical codes’ are not too broad or too specific.

For example, ‘migraine’, ‘headache’ and ‘head discomfort’ were all grouped under the clinical code called ‘Headache’. We selected this clinical code first by discussing it with clinicians and researchers who are experts in depression.

Then, with the help of a charity called McPin Foundation, we worked closely with people who have taken antidepressants to ensure that both the list of adverse events and the clinical codes were meaningful and as easy to understand as possible. We created two lists of adverse events and clinical codes – one for use by patients and one for people working in clinical practice such as doctors, pharmacists, and nurses.

Taking the lists of adverse events and clinical codes, we created a web-based, free ‘dictionary’ of terms that can be used by researchers, clinicians, patients and others. This is to help people make better shared decisions with their healthcare clinician about the prescribing and use of antidepressant medications.

The same dictionary will also be used by researchers and pharmaceutical companies, so they can collect, share, and present data about the adverse effects of antidepressants in a clear and meaningful way.
