# Supplementary material for: An online evidence-based dictionary of common adverse events of antidepressants: a new tool to empower patients and clinicians in their shared decision-making process
Source: BMC Psychiatry. 2024 Jul 25;24:532. doi: 10.1186/s12888-024-05950-6 (PMC11270875; doi:10.1186/s12888-024-05950-6)
Supplement: Supplementary file 2 — Supplementary Material 2. [file 12888_2024_5950_MOESM2_ESM.docx]

**
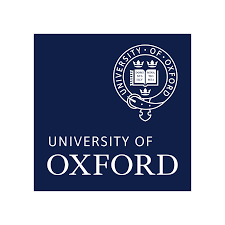

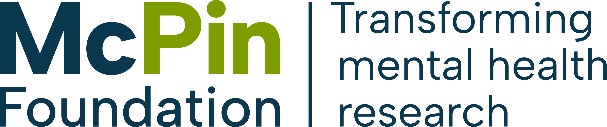
**

**Defining harms of antidepressants in depression: a free, online evidence-based dictionary of common adverse events that matter to patients and clinicians.**

**PARTICIPANT INFORMATION SHEET – FOCUS GROUP PARTICIPANT**

**Your Privacy**

**What are my choices about how my participation and how my information is used?**

There is no obligation to participate in this focus group. You can ask questions about the aims and other aspects of the focus group and the wider project before deciding to take part. If you do agree to take part, you may withdraw yourself from the focus group at any time by letting the McPin team know. You do not need to give a reason, and there will be no negative consequences of your decision. After the focus group takes place, data will be pseudonymised. Once pseudonymised, any data we have gathered will be included in the analysis even in the case that you would like to withdraw, because we would not be able to locate/withdraw your particular data. We will write our reports in a way that no-one can work out that you took part in the focus group.

Further information about your rights with respect to your personal data is available here: McPin privacy policy: <https://mcpin.org/privacy-policy/> , and Oxford University privacy policy <https://compliance.admin.ox.ac.uk/data-protection-policy>.

**Will the results of the focus group be published?**

When the project is finished the findings of the focus group will be published through journal articles, reports, presentations and/or conference papers. You will not be able to be identified in any written or verbal reports from the project. We would be happy to discuss the results of the project with you and to send you a copy of the published results once the study is completed if you would like.

**Are there any benefits in taking part?**

We hope that the information gathered from the focus group will help us understand more about patients’ understanding of the meaning, importance and interpretation of adverse events of antidepressants in their own lives. We also hope to identify opportunities to improve how patients could discuss adverse events with their healthcare providers, and to create a patient-friendly free web-based dictionary of adverse events. There will also be financial compensation provided by the McPin Foundation.

**Are there any potential risks in taking part?**

The risk involved in taking part in the focus group is that it may raise topics you may find difficult to talk about. You will be able to voice any concerns before, during or after the focus group, and are free to stop at any point should you find it too difficult to start or continue. If we are concerned about your safety, we would sign-post you to services as needed.

**Who is organising and funding the project?**

The project is being paid for by Angelini Pharma, a pharmaceutical company focused on improving our understanding of and developing more effective treatments for mental health conditions. The Principal Investigator of the study is Professor Andrea Cipriani (Professor of Psychiatry at the University of Oxford Department of Psychiatry; Lead of the Oxford Precision Psychiatry Lab). The study is being conducted by members of the Oxford Precision Psychiatry Lab in collaboration with the McPin Foundation.

**Who has reviewed this project?**

This study has been reviewed by Research Governance, Ethics & Assurance team at the University of Oxford, who have confirmed that no formal ethical approval is required.

**Who do I contact if I have a concern about the focus group?**

If you have a concern about any aspect of the focus group, please contact Senior Public Involvement in Research Officer at McPin, Roya Kamvar at [roya.kamvar@mcpin.org](mailto:roya.kamvar@mcpin.org) and Researcher at the Oxford Precision Psychiatry Lab and University of Oxford Department of Psychiatry, Dr James Hong at [james.hong@psych.ox.ac.uk](mailto:james.hong@psych.ox.ac.uk).

**How will we use information about you?**

We will need to use information about you for this project. However, this information will be pseudonymised for any publication arising from the project. People who do not need to know who you are will not be able to see your name or contact details. We will keep all information about you safe and secure.

**Where can you find out more about how your information is used?**

All data processed will be processed in line with the Data Protection Act 2018 and GDPR (General Data Protection Regulations). Data collection will also comply with the Common Law Duty of Confidentiality. The University of Oxford and the McPin Foundation are the Data Controllers and are responsible for looking after your information and using it properly.

Data protection regulation requires that we state the legal basis for processing information about you. In the case of research, this is ‘a task in the public interest.’ You can find out more about how we use your information in the McPin privacy policy: <https://mcpin.org/privacy-policy/> and Oxford University privacy Policy <https://compliance.admin.ox.ac.uk/data-protection-policy>, or by contacting the McPin team.
