# Supplementary material for: An online evidence-based dictionary of common adverse events of antidepressants: a new tool to empower patients and clinicians in their shared decision-making process
Source: BMC Psychiatry. 2024 Jul 25;24:532. doi: 10.1186/s12888-024-05950-6 (PMC11270875; doi:10.1186/s12888-024-05950-6)
Supplement: Supplementary file 3 — Supplementary Material 3. [file 12888_2024_5950_MOESM3_ESM.docx]

**
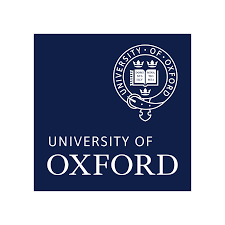

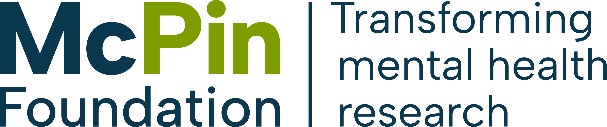
**

**Defining harms of antidepressants in depression: a free, online evidence-based dictionary of common adverse events that matter to patients and clinicians.**

**PARTICIPANT PRE-READING – FOCUS GROUP PARTICIPANT**

**Introduction**

All medications can have unwanted effects. For example, a dry mouth. Some effects are more serious than others. Within a research study these are known as ‘adverse events’. These adverse events may, or may not, be due to the medication being studied. But it is important to record everything in case it is due to the medication.

When we looked at the research data on antidepressants, we found that there were hundreds of different ways in which adverse events were reported. With the help of a charity called McPin Foundation, we started working closely with people who have taken antidepressants to ensure that the way we describe adverse events and group them are meaningful and as easy to understand as possible (these groups are called ‘clinical codes’). We are creating two lists of adverse events and clinical codes – one for use by patients and one for people working in clinical practice such as doctors, pharmacists, and nurses.

Taking the lists of adverse events and clinical codes, we are creating a free, web-based ‘dictionary’ of terms that can be used by researchers, clinicians and patients. This would help people better understand the information they need to make shared decisions with their healthcare clinician about the prescribing and use of antidepressant medications.

**What is an adverse event (as opposed to an adverse reaction, or ‘side effect’)?**

An adverse event could be any unwanted symptom we might have after starting a treatment, but it might or might not be caused directly by the treatment (we might have had the adverse event even if we had not taken the medication).

An adverse reaction is a symptom that we think is caused directly by the treatment. This is often called a ‘side effect’ in everyday language.

**Adverse Reaction**

Unwanted symptom we think is a **direct cause** of the treatment.

**Adverse Event**

**Any** unwanted symptom which could have **any** **cause, but need to report in case it is caused by the treatment.**

**Examples:** Below, we have provided examples with fictional characters to illustrate examples of adverse events or reactions.

**Adverse Event**

*Lucia suffered occasional headaches for some years. She started taking antidepressants 6 months ago, and her headaches have gotten notably worse, more painful, and more frequent. She also developed a skin rash in the second month. She has experienced headaches for a long while, and has been very stressed in the past 6 months, so is unsure if the symptoms are linked to the medication.*

**Adverse Reaction**

*Martin has only experienced nausea a few times in his life. He started antidepressants 3 weeks ago, and in the first week experienced intense nausea. It wore off after the first week. Martin said it was a difficult experience even though his GP warned him this might happen, but he was relieved when it stopped.*

**Why do we focus on antidepressants?**

Antidepressants are widely prescribed (over 17% of adults in the UK are prescribed antidepressants). They are used in a range of conditions including anxiety and chronic pain, but are most commonly used to treat depression. Depression is a mental health condition experienced by up to 18% of people during their lifetime. People with depression may experience different symptoms depending on the individual. These symptoms may include persistent low mood, loss of interest in activities they once found pleasurable, tiredness and/or not sleeping well. Depression can hugely impact a person’s life, like making them feel more isolated and struggling with everyday life, but everyone is different.

**Aims of the focus group**

- We would like to hear from you about your experiences of taking antidepressants.
- Any adverse events you may have had or other unwanted symptoms before, during or after taking them.
- We would also like to hear about your views of what might be most helpful to think about when exploring ways to improve the communication of adverse events.

**Sharing Your Experiences**

Please note that you are not obliged to disclose any detail about your personal experiences, and we do not want you to share personal details (such as your address), but we will be asking about your perspective from your lived experience as relevant to the project.
