# Supplementary material for: An online evidence-based dictionary of common adverse events of antidepressants: a new tool to empower patients and clinicians in their shared decision-making process
Source: BMC Psychiatry. 2024 Jul 25;24:532. doi: 10.1186/s12888-024-05950-6 (PMC11270875; doi:10.1186/s12888-024-05950-6)
Supplement: Supplementary file 4 — Supplementary Material 4. [file 12888_2024_5950_MOESM4_ESM.docx]

**Supplementary file 5: Latest Code List, has been uploaded to the Open Science Framework (OSF) website and can be found and viewed/downloaded at the private link below:**

<https://osf.io/2v8gr/?view_only=73c9d20b8caa41acbb371ce58a1dc203>
