# Supplementary material for: An online evidence-based dictionary of common adverse events of antidepressants: a new tool to empower patients and clinicians in their shared decision-making process
Source: BMC Psychiatry. 2024 Jul 25;24:532. doi: 10.1186/s12888-024-05950-6 (PMC11270875; doi:10.1186/s12888-024-05950-6)
Supplement: Supplementary file 5 — Supplementary Material 5. [file 12888_2024_5950_MOESM5_ESM.docx]

**Supplementary file 6: Demographics of Focus Group participants**

| Focus Group April 24^th^ 2023 Demographics | | | | | | |
| --- | --- | --- | --- | --- | --- | --- |
| **Age** | 33 | 69 | 34 | 28 | 43 | 62 |
| **Gender** | Female | Male | Female | Female | Female | Female |
| **Ethnicity** | White, British | White British | White- Other | Bangladeshi | White British | White British |

Note that 8 people is an average to above-average total number for a focus group (see for instance the reference (1) below). Whilst the focus group is mostly white, the demographic data excludes two (out of the eight) participants who did not contribute this information. Furthermore, this focus group was not the extent of lived experience involvement, with others involved including the McPin facilitators with lived experience as well as the Lived Experience Advisory Panel (LEAP) recruited by McPin (please refer to Figure 2 and Methods in the main text. Other aspects of diversity not covered here include, for instance, sexual orientation. Advice from McPin representatives, who represent an organisation with diversity and inclusivity as key priorities, were sought and incorporated throughout the project.

**Reference**

1. Henshall, C., Cipriani, A., Ruvolo, D., Macdonald, O., Wolters, L., & Koychev, I. (2019). Implementing a digital clinical decision support tool for side effects of antipsychotics: a focus group study. *Evidence-based mental health*, *22*(2), 56–60. <https://doi.org/10.1136/ebmental-2019-300086>
