# Supplementary material for: An online evidence-based dictionary of common adverse events of antidepressants: a new tool to empower patients and clinicians in their shared decision-making process
Source: BMC Psychiatry. 2024 Jul 25;24:532. doi: 10.1186/s12888-024-05950-6 (PMC11270875; doi:10.1186/s12888-024-05950-6)
Supplement: Supplementary file 7 — Supplementary Material 7. [file 12888_2024_5950_MOESM7_ESM.pdf]

Project: Defining harms of antidepressants in depression: a free, online evidence-based dictionary of common adverse events that matter to patients and clinicians
Document: Full thematic write-up
Date: 19 Aug 2023

Table of Contents

Theme 1: The Concept of Adverse Events/Reactions and Information about Adverse Events of Antidepressants........2
Definition........2
Codes........2
Summary of data........3
Points for further consideration........8
Theme 2: Shared decision-making (SDM)........9
Definition........9
Codes........9
Summary of data........11
Points for further consideration........19
Theme 3: Personalised care and facilitators, barriers and challenges to its delivery........21
Definition........21
Codes........21
Summary of data........22
Points for further consideration........29
Theme 4: Usefulness and acceptability of information in an online resource........31
Definition........31
Codes........31
Summary of data........32
Points for further consideration........35

Theme 1: The Concept of Adverse Events/Reactions and Information about Adverse Events of Antidepressants

Definition

This theme contains codes pertaining to the usage of the concepts of adverse events (AEs) and adverse reactions (ARs), the usage of language in discussing them, details about AEs that should be collected and communicated, and issues in distinguish AEs versus ARs (medication-related effects) of antidepressants.

Codes

Language of the concept of adverse events and reactions

- Uncertainty regarding regulatory terms (‘adverse event’ and ‘adverse reaction’)
- Understanding of the definition of ‘adverse event’/’adverse reaction’
- Describing the concept of adverse events

Details about adverse events (AEs)

- General need for more detailed information
- Breadth and specificity of AEs
- Symptom-based description of AEs
- Emotional and functional impact of AEs
- Seriousness of AEs
- Frequency of AEs
- Prevalence of AEs

- Likelihood of AEs
- Dose-dependence or -independence of AEs
- Duration and/or severity of AEs
- Relatable description of AEs
- Weirdness or randomness of AEs
- Lasting impact of experience around AEs
- Burden of AEs
- Expected versus unexpected AEs

Adverse events versus adverse reactions

- Pre-existing, new or change in a phenomenon (e.g., symptom)
- Persistent symptoms
- Timing of AE
- History and predictability of AE
- Experience during dose changes (titration versus tapering)
- Uncertainty in attributing causality (i.e. antidepressant-related or not antidepressant-related)
- Uncertainty in interpreting an experience as therapeutic or adverse
- Rationalising one's experience

Summary of data

Participants viewed the concepts of adverse events/adverse reactions as important and relevant to their care but expressed uncertainty about the usage of the terms in real world practice. This was expressed in both implicitly and explicitly. Five out of the nine participants expressed implicit

uncertainty, embedded in their questioning of the definitions and utility of the concepts. This was found at the beginning of the focus group (***"So just to double check, adverse events is the same as side effect, isn't it? It's just a different way of calling it?"*** **Participant 6, line 116**) as well as toward the end of the focus group where they asked for reassurance that the content of the discussion was within the scope of AE/ARs (***"I was also finding that through this whole conversation that I've actually been a bit confused about whether we were talking about side effects or events…"*** **Participant 8, line 637**). This was accompanied by an explicit statement that the terms are not relevant to 'most people', highlighting the importance of the real-world relevance of terminology: ***"I supposed the word adverse events, I don't know if that's going to mean a lot of things to quite a lot of people…so most people would talk about side effects…"*** **(Participant 5, line 612)**. Hence, in addition to the language used to describe specific AEs (i.e., to characterise the adverse experience associated with antidepressants), the language used to bring up the topic of tolerability in a clinical encounter must be appropriate and patient friendly. Expression of uncertainty, however, should not be confused with inability to understand relevant concepts, as understanding was demonstrated in some cases: ***"Focusing on events, I've been a lifelong sufferer of headaches. I still have them and I take medication for headaches, for migraines but they had become worse whilst I've been on antidepressants so there's a crossover I guess from event to reaction or both I guess."*** **(Participant 3, line 208)**

Participants referred often to the need and desire for more details to describe aspects of the adverse experience that were important to them. This expression ranged from a general need to contextualise their experience using details **(*"I think the more information about those real details about side effects, for different people with different lives and needs, you would be able to compare and contrast much more."* Participant 4, line 865)**, to more specific dimensions they felt were important to characterise. Specific aspects included:

- breadth and specificity of terms used to describe AEs: "***You can go from feeling nervous to having feelings of panic. I think they're very different feelings. I think it's actually really useful to have quite a few different words to describe what that might feel like." (*Participant 8, line 564)**
- distinguishing symptom-based versus non-symptom-based (emotional and functional impact of AEs): ***"it feels like what people are describing are quite matter of fact"* (Participant 8, line 278, referring to description/characterisation of specific symptoms)**; ***"So most people would talk about side effects and also for me, it would be more like life change as well which could include a life event or a change in circumstances rather than maybe adverse events."* (Participant 5, line 615); *"But the intensity, I wonder whether you could actually maybe describe it more as the impact it might have on your day to day life or the things that you need to do might be a way which might be relevant to more people."* (Participant 8, line 786)**
- potential seriousness (Note this must be distinguished from the regulatory definition): ***"if you understand the likelihood or the seriousness of it, it would also be one more thing you can bring to the GP so that you can be more of an equal in that shared decision making about your own health."* (Participant 12, line 604)**
- frequency of specific AEs: ***"I've also had really vivid dreams every night since I've been on Sertraline"* (Participant 11, line 268)**
- prevalence or 'commonness': ***"side effects which aren't very common to experience"* (Participant 8, line 755)**
- likelihood (see above quote from Participant 12, line 604)
- dose-dependence versus -independence: ***"Whether there's anything around that as well, about dosage, I don't know whether any of that will be included because that can***

definitely make a change. So long as you're okay on the medication but once you start to up the dosage, the side effects could be a lot more." **(Participant 5, line 848)**

- duration and severity: ***"So obviously its severity and longevity in terms of the consequences would be helpful in deciding to take it."*** **(Participant 3, line 709);** ***"I think it's really helpful to know those things, especially when you're starting the medication. I think it really helped me to know the intensity and how long it would last for so that I would stick with the medication. If I hadn't known, I don't know if I would have wanted to continue with that. So yes, it was really beneficial for me."*** **(Participant 11, line 716)**
- relatability of description: ***"In my mind, I was thinking it's a bit like, I love gardening and when you buy a plant at the garden centre, it has a little symbol and it will maybe say how big it grows and what kind of position it needs to be. So I was imaging it a bit like that for the various effects that would just give you…"*** **(Participant 4, line 804)**
- acknowledgement of the 'weirdness' of the experience: ***"I just remember I could eat, eat, eat. It was as if there was no, it was like bottomless, I didn't have an end there. It was just bizarre."*** **(Participant 6, line 329)**
- the lasting nature of the impact: ***"I can really relate to what both [Name] and [Name] said, especially the bit about the things that, the emotions that they deal with, the physical problems. So for example three things that I remember very clearly was skin rash…"*** **(Participant 6, line 324)**
- overall burden of AEs: ***"Is Citalopram the entry level antidepressant that has the fewest side effects and they start you off on that? Who knows."*** **(Participant 8, line 891)**
- expectedness versus unexpectedness: ***"…to understand what might be considered a normal symptom"*** **(Participant 12, line 992);** ***"That's what just feels so weird about all of this, is that some of this stuff feels so random and you wouldn't anticipate it to be impacted but it is."*** **(Participant 8, line 1010)**

Finally, participants expressed concern about – hence further emphasising the clinical importance of – differentiating AEs versus ARs. There was a clear desire to rationalise and interpret their experience, important for its own sake as well as to better formulate their thoughts about what to do or discuss next with the clinician and manage their own expectation and behaviour (i.e., tolerance and adherence) appropriately: ***"if I know why something is happening then I can maybe rationalise it and think, "I can't hear things properly or everything because this is what is happening to my brain. Is that rational or is that just this weird, kooky side effect?"*** **(Participant 8, line 1008).** The factors they felt were relevant to making the AE-AR distinction were consistent with reasoning used by clinicians. These included whether phenomena (i.e., symptoms) were pre-existing or new-onset or whether there was a change; whether symptoms were persistent; symptom timing (e.g., early versus later timing, and differences in the time of onset of specific AEs; the history and predictability of previously experienced AEs; and relation to dose changes (e.g., titration versus tapering). Separately, participants expressed specific factors that make it more difficult to make the AE-AR distinction, for instance, temporal gap **(*"[Migraines] could come every couple of months. So it's thinking, "Is it happening now because I've always had it or is it happening now because of the medication?"*** **Participant 12, line 223**) or overlap with symptoms of depression **(*"[disturbed sleep was a] symptom of my illness when I wasn't medicated. So again, sometimes it could be a sign that my illness is bad at that time but also I think the medication is having an effect."*** **Participant 4, line 242).** This uncertainty of casual attribution was associated with distress, confusion and self-doubt, and generated a practical need to translate understanding to decision-making about their treatment. In a special case, participants also expressed uncertainty in interpreting an experience as an AE/AR or as an expected part of a therapeutic response: ***"I wasn't feeling low but also I wasn't feeling my bubbly self. There was just numbness which at the time I was like, "This feels good," but actually it wasn't real life."*** **(Participant 6, line 334)**

Points for further consideration

- The nature of data capture for concepts relevant to AEs, may relate also to how they are best presented/communicated to them.
- Semantic variation is an acknowledged challenge in the AE literature as well as an opportunity. There must be a balance of language that validates, as well as the need to be able to use this information for greater public benefit (research that is clinically and pharmacologically meaningful).
- The use of language and types of language (e.g., jargon, lay language, etc.) are particularly complex in the AE field due to the wide range of both expected and unexpected phenomena which are not well studied.
- The conceptual apparatus for symptom-monitoring and interpretation should be co-created with those whose experience are being subject to data capture.
- Real-world relevance is important for both the subjective validation and objective usefulness of data on symptomatology.
- The use of language may either help with the rich expression of subjective experience, or if inappropriately used, subdue them due to the perception or impact of pre-existing stigma associated with antidepressant treatment/AEs.
- Because of the diversity of experiences associated with AEs, all stakeholders including patients, clinicians, developers, purchasers, adopters and evaluators of clinical decision-making tools, should take extra caution and co-implementation is likely the best approach.

Theme 2: Shared decision-making (SDM)

Definition

This theme contains codes pertaining to the role of shared decision-making (SDM) and facilitators and barriers to SDM in antidepressant treatment of depression.

Codes

Clarifying the personal therapeutic goal for SDM

- Personal therapeutic goal
- Most important symptoms of depression
- Importance of listening to the individual
- Personal priority or interest
- Multidimensional aims of therapy
- The clinician's therapeutic goals and concerns
- The patient's therapeutic goals and concerns

Interactions with the healthcare professional (HCP) and SDM

- Interaction across healthcare disciplines
- Importance of trust
- Importance of relevant clinical expertise
- Emotions around the clinical interaction
- Balance of power in the clinical interaction
- Importance of setting expectations through good communication
- Modes of communicating and digesting AE information

- Importance of discussing AEs
- Importance of discussing options
- Importance of communicating preferences

Information-seeking contexts for SDM

- First trial of antidepressant
- Switching antidepressants
- Starting an antidepressant
- Stopping an antidepressant
- Shift in healthcare system
- After long-term treatment

Perceived barriers or challenges to providing care based on SDM

- The right information at the right time
- The complexity of adherence
- Impact of time and quality of discussion
- Health care systems-related factors

SDM and enhancing patient autonomy

- Importance of informed decision-making
- Importance of patient education
- Helping people help themselves (Finding information and seeking help)
- Validation and advocacy

Summary of data

For decision-making to be collaborative and effective, there needs to be clarification of the individual's therapeutic goals (i.e., goal directed actions require a rationally established goal which is communicated/shared between relevant parties): ***"in a sense, it is values based, it depends what you want to get from the medication…"*** **(Participant 12, line 922)**. The importance of establishing a personal therapeutic goal was distinguished from, but related to, specific information to ask the patient about and the approach/attitude required to clarify or define the goal. For instance, understanding what symptom(s) are the most important to the individual requires specific information-gathering questions **(*"What symptoms of the depression itself were most important because it's more than just mood. It's also the way it affects the way you think."* Participant 12 line 913)**, and the approach/attitude required to achieve this involves listening to the individual's ideas and concerns **(*"…it's really important to listen to each person…"* Participant 12, line 923).** Additionally, personal priorities and interests may deviate from immediate or routine clinical needs but may be of equal importance in the person's conception of her/his ideal state.

The establishment of a clear therapeutic goal does not necessitate that the aim of eventual therapy should be one-dimensional. One participant explicitly mentioned more than one symptom she/he had wanted to improve during antidepressant therapy and implied that these (symptom-based) priorities could also be 'ranked' in importance. ***"I'm much more willing to accept this symptom or the medication not working in this way as long as this part is resolved," because there's a lot of things you're hoping to achieve from these medications and not just one thing."*** **(Participant 12, line 923).** Furthermore, participants' description of their experience revealed tensions that may arise between the patient and clinician when one or more elements of SDM are insufficiently met:

> ***"…the fact that they made us stay in a 72 hour hold to make sure that there were no adverse reactions [to the antidepressant, bupropion]. So as a family, it makes you feel like you're doing something wrong when, in reality, you are doing something right for yourself by taking a medication."*** **Participant 9, line 449**

Participants revealed a variety of interactions with healthcare professionals (HCPs) in various disciplines, highlighting both good and not-so-good experiences. The discussion brought into focus the extent of multi-disciplinary collaboration needed to effectively deliver SDM-based care. To strike an acceptable balance between benefit and risk of treatment, SDM requires appropriate sources of information to be gathered and synthesised. Pharmacists were perceived to be helpful in providing expert medication advice, in addition to information obtained during the 'primary encounter' (i.e., a primary encounter is defined here as the encounter with a clinician who would ultimately make prescribing decisions, most typically GPs):

> ***"I'm lucky that my GP is specifically interested in overprescribing and withdrawal."*** **Participant 8, line 697**

> ***"…really important role for pharmacists attached to GP surgeries – I am lucky that my GP surgery have 2 full time pharmacists…they do all the reviews and supported me with increases and decreases in medication doses"*** **Zoom chat, line 51**

However, not all participants had ready access to pharmacists' advice for support during antidepressant treatment. This highlighted the importance of clinical expertise that is readily accessible, for the right person in the right place at the right time, and relevant to the clinical topic of SDM at hand. In this case, multidisciplinary clinical expertise was required to facilitate the effective

clinical assessment of and interaction with people with major depression, and to provide support during antidepressant treatment. The relevance of clinical expertise became clearer, as participants revealed what must be discussed more clearly (i.e., the Content) in the SDM process: different therapeutic options and their efficacy and tolerability profiles (information about AEs), individual preferences regarding AEs and various dimensions such as severity, duration and time course of effects. Taken together, these elements would inform their decision about antidepressant therapy.

The delivery of information during the SDM process involves not only the 'what', but also the 'how'. For instance, a leaflet that accompanies a box of medications is one form of communicating AEs in written form. This form of communication is, however, difficult to contextualise, interpret and for some people, to access (e.g., for those with visual impairments or dyslexia). Even if one could theoretically read all the information, one participant highlighted how she/he often would not, resulting in unused information (Information Waste): ***"You know like when you get given a form, you get your box of tablets and it has all the side effects and you read up to the mid-way which is the common. I hadn't ever really gone into the rare, the warning or whatever."*** **Participant 8, line 284.** One of the aims of SDM is to set appropriate expectations for both patients and clinicians and effective communication is required for this. Participants described how the quality of this expectation-setting was generally better achieved through empathetic person-to-person communication of relevant and sufficiently detailed information (the Content). Ultimately, participants emphasised the importance of trust in the clinician, and trust was identified to be a function of the following:

> <u>Accuracy of the Content </u>(which, when delivered directly by a clinician, was associated with clinical expertise): ***"It was amazing because it was exactly how [the GP] said it would work. So yes, I had a very good experience in that sense."*** **Participant 6, line 741**

Quality of communication*: **"[the clinician] had sold it in a really blasé way, 'You might feel a little bit wobbly. You might feel a bit thirsty,' it was totally played down."*** **Participant 8, line 287**

Quality of the relationship: ***"…whether you've got a longer term relationship with a GP…I, for the first time in my life, have got one named GP that I see all the time and that's really helped."*** **Participant 8, line 692**

Optimisation of the various aspects of the SDM process around antidepressant prescribing would also be critical to the clinical and ethical quality of the patient experience, given the intensity and variety of emotions expressed by the participants. These feelings were diverse. They included feelings of having been heard (reflecting empathy), self-doubt, denial, feeling judged, overwhelmed, feeling prepared or unprepared, worried, not informed and perhaps most strikingly (as it is directly against the ethical tenets of SDM and indeed of modern healthcare), feeling forced into a choice.

Feeling of having been heard: ***"[The clinician] was really good … She was very caring."*** **Participant 6, line 732**

Self-doubt: ***"I thought was it [i.e., starting to feel suicidal] the nature of the medication perhaps not working so well with me."*** **Participant 5, line 250**

Denial: ***"I was actually told that the things I was experiencing wasn't related [to the antidepressant] but I knew it was."*** **Participant 8, line 701**

Feeling judged: ***"…it was just a clear cut, 'No we cannot prescribe that for you because we don't do that here. You're not a smoker so why do you need this?"*** **Participant 9, line 424**

Feeling overwhelmed: ***"…I was on the absolute cusp of coping emotionally each day. So then to also have all of these other physical things to manage was nearly impossible."*** **Participant 8, line 294**

Feeling prepared: ***"I think I was prepared for that because I had a long session about that [the side effects]."*** **Participant 11, line 405**

Feeling unprepared: ***"I think I was totally naïve into the process of what starting this medication would be for me."*** **Participant 8, line 289**

Feeling worried: ***"My GP was mentioning the suicidal thoughts that might be occurring. So I was really worried about."*** **Participant 11, line 402**

Feeling uninformed: ***"I just feel like would I eat something that I didn't know what was in it or didn't know what it was going to do to me."*** **Participant 8, line 1005**

Feeling forced into a choice: ***"when I first started antidepressants, I was breastfeeding and I wasn't given a choice. They said, 'You have to take sertraline,' that was it. I wasn't given a choice even though I didn't find it effective."*** **Participant 12, line 909**

Specific information-seeking contexts were identified, including starting an antidepressant, switching antidepressants, stopping an antidepressant, moving to a different healthcare system (e.g., US to

UK), and after long-term stability on an antidepressant. These various Scenarios are important because some aspects of the SDM process discussed above may be modulated by the specific context, and each information-seeking context represents a decision-point in a patient's journey where prior SDM experiences could be evaluated and improved upon.

Participants revealed key barriers or challenges to receiving/delivering SDM-based care, these could broadly be divided into individual level and systems-level factors. At the individual level, a person might not be aware of available options (or even the possibility of asking for alternative options); receive incorrect/inaccurate advice; be uncertain of when/how to seek help; or experience stigma about seeking help: ***"I'd be worried about people feeling they have to be 'bad enough' and that feeds a feeling of stigma"*** **Zoom chat, line 49.** These affect a participant's ability to receive the right information at the right time. Second, participants implied that 'adherence' was a complex concept which represents a conceptual challenge to what may constitute SDM. For instance, even if a patient is aware of alternatives, knows how to seek advice, and is willing and able to ask for advice, she/he may adhere because coping with withdrawal symptoms may be too difficult. In other words, the participant cannot choose an option of 'no treatment', even if she/he could realistically choose between different antidepressant options. ***"In my case, I found it so hard to withdraw that I'm just on a medication just because it's easier to stay on it than to come off. That's not the right place to be in…"*** **(Participant 8, line 901).** Third, participants highlighted the impact of time and the quality of discussion had with HCPs on feeling they had come to a decision through a SDM process: ***"…being able to secure 20-25 minute long appointments. That was really when we discussed the medication…I had to pay for [the private consultation] because there's just no way to have these conversations right now with the way that GP practices are."*** **(Participant 9, line 429)**. Whilst the importance of time was mentioned frequently, this probably reflects the need for a higher-quality SDM process, in which time is a practically but not theoretically required resource. One could

imagine having a 30-minute session with a clinician where the personal therapeutic goal is not discussed or established, or information is presented in a way that cannot be easily understood or processed by the patient.

The time factor represents an 'intermediate' barrier to SDM, reflecting both individual and systems-level challenges. At the systems level, participants distinguished between their experiences of the UK and US healthcare systems, describing different standards of practice, and highlighting the limitations of over-reliance on guideline-based practice. For instance, regarding bupropion, ***"it's so commonly prescribed there [in the USA] for the things that I was experiencing, that it seemed like a natural fit."*** (Participant 9, line 415). However, after moving to the UK, ***"during the pregnancy, it was really scrutinised that I was on this medication so there was constant advocating for myself to say that this was right, it is normal in the States…"*** (Participant 9, line 443). This systems-level challenge is associated with geographic, socio-politico-cultural variations in mental healthcare practice.

Second, some participants made a distinction between their experiences of public and private healthcare services:

> ***"I would distinguish again between the public health services and private. So my experience with GPs and psychiatrists from the NHS is that they would not really have a great deal of time to go into any great detail about these things. Whereas in a private sector they would basically."*** **Participant 3, line 673**

It may be helpful to consider how and why certain sector-specific services were perceived as of greater or lesser quality. This could involve, for example, investigating operational, institutional, cultural or other factors affecting the ability of individual actors in these systems to deliver SDM and

other processes relevant to delivering high-quality care. For instance, one participant described the following experience:

> "***I've had a slightly different experience than [Name]. I've managed to find a GP that does have experience and interest and does give me time. But my last wait for my GP was over an hour so he runs behind. That's how I get time with him, is because he is always running…"*** **Participant 8, line 678.**

Third, participants revealed a contrast between continuous and discontinuous care. The structure and distribution of service infrastructure and processes that engender continuous or discontinuous care affected in many cases the participant's ability to form a long-term relationship with the clinician. The value of this relationship lied in the ability to discuss and make more collaborative and personalised decisions about antidepressant treatment: ***"…great when get the same GP and understands"*** **(Zoom chat, line 72); *"…if you were lucky enough to have [a discussion] with a doctor about which medication you might prefer."* (Participant 4, line 861).**

Finally, SDM is driven by autonomy-enhancing processes, therefore efforts to improve SDM would involve efforts to promote individual autonomy. Education regarding antidepressants and adverse events would help individuals to make a more informed choice, because ***"if you, as the patient, one as the patient, is aware of the full impact of taking that medication, then you can make an informed decision about whether to take it."*** **(Participant 3, line 712)**, and empower individuals to learn about and better manage their health. As one participant believes, ***"…in the end, you have to learn for yourself. You learn, by stepping up through medication, often how sensitive you are or not to the effects."*** **(Participant 4, line 813).** Importantly, participants emphasised the central role of validation and advocacy in their treatment journey, for instance by being able to express what are

important to them in their treatment. The ability to advocate for themselves was affected not only by depression but also by the nature of interaction and decision-making with the clinician.

Points for further consideration

- Multidisciplinary expertise is highlighted as important in primary antidepressant therapy, but this capacity to deliver this is variable across services. Clinicians may benefit from additional training and support in their work in the management of depression, especially in the community.
- Expectation-setting is important in adherence and satisfaction with care, but it is challenging to do this well in practice. This requires the delivery of accurate and contextually appropriate evidence as well as advanced communication skills and ability to build rapport. A digital support tool must consider these multiple facets. Future research should investigate effective ways to set and measure expectations, and the association with adherence, satisfaction and clinical outcomes.
- Guidelines are useful, but over-reliance on them results in impact on quality of care, patient satisfaction and the quality of rapport.
- Simply choosing to continue an antidepressant should not be assumed to be an autonomous choice. Reasons for choosing to continue to take a medication is just as important as reasons for choosing to stop.
- Services require increase in capacity as well as more efficient and creative ways to better use distributes resources. This will become more important given staff and budget constraints within the NHS.
- We should not under-estimate the patient's desire or ability to seek information relevant to their antidepressant therapy. However, they may benefit from guidance like in any field of education. Effective education programmes should include information on where to seek

information, how to access them, when (in what contexts or scenarios), and support their conversation with a clinician.

- Patients may desire greater ownership of and engagement in their own care but may need support in making sense of relevant data and how to do work in partnership with their clinician.

Theme 3: Personalised care and facilitators, barriers and challenges to its delivery

Definition

This theme contains codes pertaining to the personalised care of people with depression and facilitators/barriers/challenges to a personalised approach.

Codes

Understanding the patient as an individual

- Importance of understanding at the individual level
- Acknowledging interindividual variability of adverse experience

Considering context around the AE experience to personalise care

- Individual psychosocial context
- Considering a holistic treatment approach
- Psychosocial intervention

Considering the impact of depression on the person's life and functional capacity to personalise care

- Impact on the person
- Impact on cognition

Antidepressant history (history-specific memories) and personalising care by engaging with the patient's identity

- Specific antidepressants, dose, therapeutic response

Approaches to management, personalised versus non-personalised

- Trial-and-error approach
- One-size-fits-fall (non-personalised) versus personalised
- Risk-based approach
- Measurement-based approach
- Paternalistic approach

Summary of data

Exploration of SDM (Theme 2) revealed that a key value of a longer-term, collaborative doctor-patient relationship lies in the potential to achieve better personalisation. Elaborating on the next major theme – personalised care and facilitators, barriers and challenges to its delivery – one participant stated how a clinician ***"might not know your situation or they might [not] know the medication or even what you're dealing with very well"*** **(Participant 12, line 596)**. It was implied that individual uniqueness results in uniqueness of the subjective experience of adverse events/reactions ***("I've not met another person whose had noise sensitivity or even some of the side effects I've described."*** **Participant 8, line 781**). Furthermore, there are individual differences in ability to tolerate varying intensities and durations of specific AEs (***"Some people can just stop taking the drugs and they're fine. I'm doing it over six months because I'm so sensitive to it."*** **Participant 8, line 783**; ***"…the severity, again, it's really personal, isn't it…"*** **Participant 4, line 810**). Therefore, a simple acknowledgement that each person is unique (i.e., has a distinct combination of individual-level characteristics) could be helpful in strengthening rapport and set the scene for personalisation. This helps to share the clinician's concerns about the current state of clinical decision-making and the

limitations of group-level reasoning, and to make clear the need for individual-level decision making (i.e., personalisation).

Beyond the consideration of 'internal' (biological/clinical) characteristics, most of the participants drew attention to the individual's 'external' or 'extended' characteristics. These are commonly referred to as the psychosocial context, which includes the person's life circumstances/events (e.g., bereavement, newlywed, pregnancy, breastfeeding, upcoming job interview), socio-economic status and occupation (e.g., current job requiring manual dexterity or coordination). The focus group revealed how these factors may affect several aspects relevant to personalisation, including:

- <u>*a priori* acceptability of specific AEs</u>

  being a ***"newlywed at that point so it was really important to have libido there"*** **Participant 9, line 417**

- <u>personal perception of a rational/acceptable benefit-risk ratio</u>

  (e.g., perinatal period and effect on willingness to accept a specific benefit-risk ratio and statistical uncertainties around it: ***"…there have been only two trials and they've been so small that there hasn't been any conclusive evidence that it does harm the baby by any means…I know that I would be a lot worse off if I hadn't taken it during the pregnancy."*** **Participant 9, line 452**

- <u>the optimal timing of starting antidepressant consumption, after establishing that antidepressants are clinically indicated</u>

> ***"if you've got an interview next week, you might want to think about starting them the week after…even if you're just prompted to consider when you might start taking them would be helpful."*** **Participant 8, line 770**

Participants referred to what we might call a holistic approach to management – that is, an approach that considers a range of internal and external/extended characteristics of the person to deliver multi-component, integrated interventions simultaneously. Components may include, for example, lifestyle or social interventions, programmes to address alcohol/substance dependence and additional support to optimise the person's psychological readiness before starting an antidepressant.

> ***"So for example, if you've got a weekend at home, you could perhaps start the new [antidepressant]… I think it can really vary whether you get that advice or not, whether you can be in a safe, comforting space to try them."*** **Participant 8, line 767**

> ***"…an approach was developed how to get better because by that time I was also starting to drink heavily…[the psychiatrist] changed my antidepressant regime and also encouraged me to stop drinking."*** **Participant 3, line 361**

> ***Considering the anticipated time course of clinical effects, the GP "signed me off from work. She said, 'Because of that, I'm going to sign you off for a month.'"*** **Participant 6, line 740**

In thinking about personalisation, the discussion revealed the importance of considering how depression, conceived of as a brain disorder, affects the person's ability to cope with AEs and their

consequences, and to advocate for her/himself in interactions with clinicians. This effect would manifest differently among individuals. The impact of depression and antidepressants on the person's life was therefore perceived to be mediated by - but not solely attributable to - individual symptoms of the underlying disorder or medication-related effects. One participant drew particular attention to cognitive effects because ***"certain drugs might impact different ways of thinking"*** **(Participant 8, line 1004)**: the variable degree and nature of the impact should be accounted for in personalised care. More broadly, participants felt that the clinician should consider "***the knock-on impact that [adverse events have] on your day, if you're also having to manage these things alongside daily life"*** **(Participant 8, line 282)** and that the person can be "***very unwell going on to my antidepressant"*** **(Participant 8, line 284).** This message about personalisation relates also to a point about maximising the user-friendliness (e.g., ease of use) of patient-facing resources:

> ***"I think the less work people have to do to feel like what they're experiencing is valid or real, and there's a label that they can use or point to is better than them putting it on to the person that's having those negative feelings, have to go and research or click on extra links and expand it all out."*** **Participant 8, line 573**

Throughout the focus group, participants were consistently specific and explicit about the type of antidepressant (e.g., sertraline, escitalopram, amitriptyline) that they had been on, which they made clear before going on to describe their experience. ***"I've been on sertraline and escitalopram, those are the ones I have experience with."*** **(Participant 12, line 192)**; ***"Actually many years ago I took sertraline for my nausea with it…"*** **(Participant 5, line 247)**; ***"…had other things like amitriptyline which was pretty horrendous, blurred vision, metallic taste in your mouth. That certainly was a pretty horrendous side effects there…"*** **(Participant 5, line 253).** The participants' perception of depression and the journey of antidepressant therapy was intimately tied to and centred around

'their' or 'my' antidepressant. This could be compared to how one might refer to certain conditions in the possessive, e.g., 'my anxiety', 'my mood', 'my drinking', as did participants in our focus group. In contrast, there is a general tendency to objectify diseases in other domains, such as cancer, both mentally and linguistically. Given the special place of subjective experience in mental health, it would not be unexpected to find that specific antidepressants play a central role in recollections of the experience of treatment. Indeed, participants described a profound emotional exhaustion from effects associated with antidepressant therapy:

> ***"I think my biggest one, I just feel like it's really, I suppose what we're talking about, it feels like what people are describing are quite matter of fact and we're not describing the emotional impact that having headaches and nausea and disrupted dreams and disrupted sleep has on you emotionally. So I'm interested in that bit of it as well because that's the adverse event as well, isn't it?"*** **Participant 8, line 277**

Antidepressants – whether referred to by their brand or generic names – appear to remain an integral part of the participant's journey (the lived experience of depression) and their perception of it, beyond the factual nature of antidepressants as clinical data to be ascertained in psychiatric history-taking.

> ***"…and so my history with taking the medication that I'm on currently is Wellbutrin in the States, also known as bupropion here…It's been better than anything else I've tried but here in the UK, it's often only prescribed for smoking cessation…I've experienced different things while taking it, however I would say the pros outweigh the cons."*** **Participant 9, line 84**

Personalised care could therefore be conceived to involve different facets of engagement. One facet involves an engagement of the objective individual, through the characterisation of the person's clinical profile and history (e.g., considering the person's history of antidepressants and their pharmacologic effects, including the dose and degree of clinical response). The other involves engagement of the subjective person's sense of identity that has been shaped by the impactful memories associated with their antidepressants, adverse events and unique psychosocial context.

Participants also differentiated between approaches to the management of depression. First, most participants described their antidepressant as having been selected using a trial-and-error approach, in some cases involving trials of several different antidepressants and experiencing many AE/ARs before coming to an acceptable option. There was a mixed feeling that on the one hand, participants accepted the approach because it was something that most people go through (***"I mean I know lots of people's experience is you kind of just get given something and you try it and if you can tolerate it then you probably just stay on it."*** **Participant 4, line 863**), and on the other hand, belief and hope that a personalised approach is possible: ***"I also discussed it with my therapist at the time to try and find different medications that might be a good fit but she wasn't a psychiatrist so she couldn't prescribe anything but we just discussed the different options out there."*** **Participant 9, line 419**.

One participant highlighted how she felt that her prescribing clinician just 'hoped' it would work, implying a form of decision-making which they felt was inadequate **(Participant 8, line 689)**. She referred also to experiencing a 'one-size-fits-all' or 'non-personalised' approach **(Participant 8, line 891)**. Whilst they are conceptually distinct, 'trial-and-error', 'one-size-fits-all' and 'non-personalised' approaches overlap in practical meaning. For instance, a one-size-fits-all approach often leads to the need to trial several medications (***"I've tried quite a few different antidepressants. The one I'm [on] now is one that seems to work quite well for me…"*** **Participant 5, line 251)**, based on one or more

ways of reasoning (e.g., clinical guidelines, evidence syntheses; *a priori* pharmacologic knowledge considering individual symptomatology; or non-systematic (random) reasoning in cases of desperation and lack of remaining available options).

The above were contrasted with an approach that could more effectively and efficiently find a medication that was a 'good fit' for the participant as an individual. Theoretically, one could still find the 'right' medication based on trial-and-error (as **Participant 5 describes in line 251**) or even chance. However, these were implied to be not only less efficient but also potentially damaging to the participant's experience and care, due to the emotional impact of adverse events, impact on adherence and clinical outcomes, reduced motivation to try alternative antidepressants, and impact on trust in the clinician and healthcare system. Exactly how a personalised approach would be implemented was not discussed at length. However, as outlined in Themes 1 and 2, this would require both the rigorous collection, organisation, analysis and communication of data (here, focusing on AEs/ARs) together providing individualised benefit-risk predictions, and an optimised process of SDM through which this is acted upon.

Other approaches, with some relation to those previously discussed, included measurement-based, risk-based and paternalistic approaches. Measurement-based care, as one participant felt, is not sufficient on its own: ***"I got a follow-up call asking about the…they do the scorings, the two measures, your anxiety and your depression. Some of those raised risk points so I got a follow-up call asking if I was at risk to myself. I said no. Then nothing."*** **Participant 8, line 686**. The paternalistic approach is of particular importance as it is the opposite of a collaborative, shared approach. However, it would be interesting to consider how patients would perceive and clinically respond to a treatment approach which is paternalistic but personalised; not because such an approach would be desirable in practice, but to highlight the distinctions between paternalistic care,

collaborative care and personalised care models. One participant describes how when she was breastfeeding and prescribed an antidepressant, she was told that she ***'[has] to take sertraline'*** and ***'wasn't given a choice even though [she] didn't find it effective'*** **(Participant 12, line 910)**. Here she describes two distinct components to the decision-making: (i) not having been informed of/aware of options and (ii) being told what is best for her.

Points for further consideration

- Participants are intuitively aware that they are subject to group-level decision-making. An explicit acknowledgement of this fact during clinical encounters, and the limitations of the current ways of applying evidence to practice, may help to build rapport.
- Non-personalised approaches may be associated with reduced quality of and satisfaction with care. They may also accentuate the impact of social inequalities due to reliance on self-funded care in the hope of a personalised approach.
- There are many details that patients feel are important to their decision-making in antidepressant therapy, but they may need support in thinking through these details and what further information they should seek in trying to personalise treatments.
- Paternalistic approaches to care remain in the care system. This is undesirable from an ethical perspective due to the infringement of patient autonomy and individual goals not being met. From a clinical perspective, a paternalistic approach makes personalised care either more difficult or impossible to implement.
- Clinical objectives based on 'primary endpoint'-thinking are being enforced onto patients, resulting in a misapplication of concepts outside of their intended sphere of usage. However, there are clear opportunities to implement patient-centred, personalised care by considering options, preferences and methods of communication. This will require advanced methods of engaging with the objective individual (e.g., clinico-biological characterisation of the person),

along with a profound level of engagement with the subjective individual in their therapeutic journey.

Theme 4: Usefulness and acceptability of information in an online resource

Definition

This theme contains codes pertaining to the limitations of currently available resources and explores qualities, format, content and uses of an online resource to enhance SDM/personalised antidepressant treatment incorporating AEs.

Codes

Limitations of currently available resources on antidepressants and AEs

- Current state of information

Important qualities of an online resource

- Breadth and specificity of AEs
- Accessibility and inclusivity
- Simple, clear language
- Ease of use
- Credibility
- Balance of website features
- Format of the resource: Visual format, preferences for format

Uses for an online resource

- To engage in shared decision-making

- To seek information
- To validate one's experience

Pharmacology informing AE interpretation

- Antidepressant pharmacology
- Brain mechanisms
- Dose effect information

Summary of data

The discussion touched upon on the usefulness and acceptability of information about AEs in an online resource. Comments in this domain were expected based our topic guide. Moreover, the action-oriented nature of our participants – who are embedded in the experience they described – naturally generated some thoughts on what should be considered in developing an effective and user-friendly resource. Two participants remarked on the limitations of current resources, e.g., partial availability of relevant information (***"information [about duration and severity of AEs] is kind of there to a point but not really"*** **Participant 4, line 870)** or that tools exist but not in a way that could be used to enhance communication (***"I can read and write, I can research things in theory, I have the tools at my disposal to make an informed choice but I didn't feel like I could ask for something different"*** **Participant 8, line 885**).

Some of the qualities of an online resource that participants described reflected desirable qualities of the language of adverse events/reactions discussed in Theme 1. These included:

- the breadth and specificity of AEs in an online resource: ***"I think there's a lot of benefit to show the breadth of how that issue might present in an individual."*** **Participant 8, line 576**

- the use of simple, accessible and inclusive language: ***"it's very helpful to have language that Joe public would use. When I go and see my GP, I don't talk about having gastroenter-, whatever it was, I can't even say it let alone mention it to my doctor. I would probably go in and say I have constipation. A common language that most people use is obviously helpful when you're talking about side effects or adverse events."*** **Participant 3, line 630.** (See also Participant 5, lines 613, 616 and 621)
- credibility of the research: ***"that a GP might maybe respect you a bit more if you're actually pointing to a resource and saying I'm experiencing tick, tick, tick, this, this, this for example."*** **Participant 8, line 586**

Other qualities were broadly applicable to the development of any patient-facing resource, including an online resource on antidepressant decision-making and adverse events:

- ease of use: ***"me, I find that quite easy to go to the news and I quite like that visual thing. I think to be able to check something out about it and then click on other links but that's just a personal thing, that would work quite well for me."*** **Participant 5, line 853**
- balance of features: ***"unpack the pros and cons of having those extra words there."*** **Participant 8, line 560**
- a visual format to communicate details of AEs: ***"keep some idea of the severity of either, perhaps via the little icon or smiley or something, would be helpful."*** **Participant 3, line 826;** ***"I think it would be really nice to have that visual, almost like a grid of different medications, which ones have a likelihood of having certain effects to it."*** **Participant 12, line 906**

Importantly, participants described how an online resource with the above qualities could ultimately help patients to make a shared decision about their antidepressant treatment with their prescribing

clinician. This could be done, for instance, by guiding patients on what to focus on discussing at their next healthcare appointment (***"…it is categories in a way that says this is what's serious, this is what you need to talk to your GP…"*** **Participant 12, line 602)** and to more easily or effectively compare options based on their preferences:

> ***"Something that's a real slow burn might be better for some people but other people might…have the ability to take a big hit on lots of intensity but over a shorter period…I think that comparison around the length of time you'll put up with these varying things would really help you…"*** **(Participant 4, line 867)**

Participants stressed how such a resource would be useful when seeking information tailored to their needs. Some participants mentioned explicitly the types of information they would hope to seek and find (e.g., dosage and AEs, Participant 5, line 849), whilst others mentioned that they may still need guidance in using an online resource. For instance, they may need support in identifying what information to seek in given context and how best to express their information-based concerns and preferences to their clinician **(*"So it was like what was I looking for? What symptoms of the depression itself were most important because it's more than just mood."* Participant 12, line 913).** Third, participants recognised the ability to use the resource as a way to help validate their lived experience. A research-backed, co-created resource could, for instance, help a person to realise that they are not alone in their experience or journey and that they are part of a community **(*"But I still think a gauge, to know that for lots of people it's this rough amount of time, at least you can keep the faith if you feel rubbish but you can see that for a lot of people it's a long effect. I like it."* Participant 4, line 818)**. Others felt that pharmacologic information would be useful for them to think through their own experience and try to rationalise the expected- versus unexpectedness of their adverse events/reactions: ***"…a brief description within the dictionary, within the medication***

itself on how the body uses it, how it functions within the body might be useful to anticipate symptoms even if they're not listed." **Participant 12, line 997.**

Points for further consideration

- The effective consideration of antidepressant options requires that information be presented in user-friendly ways, be available in various, easy-to-digest, accessible formats, and be organised in a way that rationally compares potential decisions and the balance of effects.
- Training and guidance would also be needed for both patients and clinicians to effectively use resources or tools to aid decision-making.
- Accessibility and inclusivity would lead to greater adoption of a patient-oriented resource, and therefore maximise its potential impact for the intended population.
- The impact of a person's condition, therapy and circumstances must be adequately considered in the design of a web-based resource or any other tool.
- Co-created resources or tools could enhance the capacity for individuals take ownership and manage their own health, with potential impact on both health and economic outcomes at individual and population levels.
- Co-created, empathy-enhancing technologies should be accompanied by effective and targeted social media and related strategies to leverage the network effect among peers with lived experience and maximise the impact of the work.
